# Combined Fungal Elicitor and Methyl Jasmonate Enhance Alkaloid Accumulation in *Catharanthus roseus* Suspension Cultures: An Integrated Molecular and Phytochemical Study

**DOI:** 10.3390/plants15193051

**Published:** 2026-10-06

**Authors:** Pham Thi Hoe, Nguyen Chi Mai, Ninh Thi Ngoc, Nguyen Tuong Van, Tran Thi Hong Hanh, Ninh Khac Ban, Vu Huong Giang, Nguyen Xuan Cuong, Le Quynh Lien, Nguyen Hoai Nam, Tran My Linh

**Affiliations:** 1 Institute of Chemistry, Vietnam Academy of Science and Technology, 18 Hoang Quoc Viet, Nghia Do, Hanoi 10072, Vietnam; hoepham93@gmail.com (P.T.H.); maiquynhhung08@gmail.com (N.C.M.); ngocninh89@gmail.com (N.T.N.); honghanh.imbc@gmail.com (T.T.H.H.); ninhkhacban@gmail.com (N.K.B.); huonggiangkc@gmail.com (V.H.G.); cuongnx@ich.vast.vn (N.X.C.); lienle@vast.gov.vn (L.Q.L.); namnguyenhoai@ich.vast.vn (N.H.N.); 2 VNTEST Institute for Quality Testing and Inspection, Lot DM10-1 Small Industry Cluster, Van Phuc Silk Village, Ha Dong, Hanoi 10000, Vietnam; vanngtg@gmail.com

**Keywords:** *Catharanthus roseus*, suspension culture, fungal elicitation, methyl jasmonate, terpenoid indole alkaloids, lignan

## Abstract

Suspension cultures of *Catharanthus roseus* provide an alternative system for studying the regulation of terpenoid indole alkaloid (TIA) biosynthesis and the production of pharmaceutically important metabolites. This study evaluated the effects of the endophytic fungal elicitor RN3 (*Chaetomium funicola*) and methyl jasmonate (MeJA) on alkaloid accumulation and TIA pathway-associated gene expression in *C. roseus* suspension cultures. Co-elicitation with 1% RN3 and 150 μM MeJA produced the highest total alkaloid accumulation (14.68 mg g^−1^ dry weight), representing a 58.9% increase over the untreated control. HPLC analysis detected catharanthine in cultures treated with 1% RN3, whereas both catharanthine and vindoline were detected after co-treatment with 1% RN3 and 150 μM MeJA. Phytochemical investigation of the co-elicited cultures resulted in the isolation of five compounds, including the previously undescribed lignan catharalignan, the alkaloids β-carboline and tryptoline, (+)-syringaresinol-β-D-glucoside, and L-thymidine. RT-qPCR analysis showed that the expression of the transcription factor genes *ORCA2* and *ORCA3* remained significantly elevated throughout the 3-day period following elicitation, whereas the tested TIA pathway structural genes showed limited transcriptional changes. These findings indicate that RN3, particularly in combination with MeJA, enhances alkaloid accumulation and modulates the transcriptional response of TIA pathway-associated genes.

## 1. Introduction

*Catharanthus roseus* (L.) G. Don is one of the most extensively studied medicinal plants because of its ability to synthesize numerous terpenoid indole alkaloids (TIAs), many of which possess important pharmaceutical activities. Among these metabolites, catharanthine, vindoline, ajmalicine, serpentine, vinblastine, and vincristine have attracted considerable attention because of their therapeutic potential [1]. However, these valuable alkaloids accumulate at extremely low levels in planta, making their commercial production challenging. Consequently, in vitro cell suspension cultures have been developed as alternative systems for studying alkaloid metabolism and enhancing alkaloid production under controlled conditions [1,2,3].

Among the strategies used to enhance specialized metabolite production in plant cell cultures, elicitation is one of the most effective approaches [4,5]. Both abiotic elicitors, such as methyl jasmonate (MeJA), and biotic elicitors, including fungal-derived elicitors, activate plant defense signaling and stimulate the biosynthesis of specialized metabolites [5,6]. In *C. roseus*, MeJA is a key signaling molecule that regulates genes involved in TIA biosynthesis. It has been widely used to enhance the accumulation of TIAs, including ajmalicine, catharanthine, and vindoline, in cell suspension cultures and hairy roots [3,7,8]. Fungal-derived elicitors have also attracted attention because they activate plant defense responses and can stimulate alkaloid biosynthesis [9]. Several studies have shown that fungal elicitation enhances the production of TIAs or their precursors in *C. roseus* cultures. For example, elicitation with *Aspergillus niger* increased vinblastine and vincristine accumulation in *C. roseus* callus cultures under specific treatment conditions [10].

Endophytic fungi isolated from *C. roseus*, particularly *Fusarium oxysporum*, also stimulated alkaloid production and, in some cases, have been reported to produce vinblastine- and vincristine-like compounds under culture conditions [11]. Our previous study showed that cell extracts prepared from the endophytic fungi *Fusarium solani* RN1 and *Chaetomium funicola* RN3 significantly increased alkaloid accumulation in *C. roseus* suspension cultures [12].

TIA biosynthesis in *C. roseus* is one of the most complex specialized metabolic pathways in plants. It involves more than 30 enzymatic steps distributed across multiple subcellular compartments [13]. Tryptophan-derived tryptamine condenses with secologanin, produced through the iridoid pathway, via strictosidine synthase to form strictosidine, the central precursor of more than 130 monoterpenoid indole alkaloids [1,13]. Representative structural genes, including tryptophan decarboxylase (*TDC*), geraniol 10-hydroxylase (*G10H*), strictosidine synthase (*STR*), strictosidine beta-glucosidase (*SGD*), deacetoxyvindoline 4-hydroxylase (*D4H*), and deacetylvindoline O-acetyltransferase (*DAT*), catalyze key steps in this pathway and are commonly monitored to assess transcriptional responses to elicitation [14,15]. The expression of these genes is regulated by several jasmonate-responsive AP2/ERF transcription factors, particularly ORCA2 (octadecanoid-responsive Catharanthus AP2-domain protein 2) and ORCA3 (octadecanoid-responsive Catharanthus AP2-domain protein 3), which are important regulators of TIA biosynthesis [16,17,18].

Previous studies have demonstrated the potential of fungal elicitors and MeJA to enhance alkaloid production in *C. roseus*. Recent studies have examined different aspects of the elicitation response in *C. roseus*, including defense-related responses, biomass accumulation, TIA-related gene expression, and alkaloid production [19,20]. However, elicitor-induced responses are highly context-dependent, and the coordinated responses of *C. roseus* suspension cultures to fungal elicitors and MeJA in terms of cell growth, alkaloid accumulation, TIA-related gene expression, and metabolite profiles remain insufficiently understood [3,21].

Therefore, this study aimed to investigate the effects of the endophytic fungal elicitor RN3 and methyl jasmonate, applied alone or in combination, on growth, alkaloid accumulation, TIA-related gene expression, and metabolite profiles in *C. roseus* suspension cultures. Cell growth, total alkaloid content, expression of selected TIA-associated genes, and representative TIAs were analyzed under different elicitation treatments. The treatment that produced the highest alkaloid accumulation was subsequently selected for phytochemical investigation to isolate and structurally identify metabolites present in the elicited culture.

## 2. Results

### 2.1. Biomass Growth

In the present study, the H01 cell line, which exhibited robust and stable growth, was selected for subsequent experiments. Based on the growth kinetics of H01, elicitors were applied on day 18, corresponding to the late exponential phase and preceding the stationary phase. The RN3 strain of *Chaetomium funicola* Cooke was previously isolated from *C. roseus* root samples by Linh et al. [12]. RN3 was used as a fungal elicitor, either alone or in combination with methyl jasmonate (MeJA), in subsequent elicitation experiments with H01 suspension cultures.

Fresh and dry weights were monitored for six days following elicitor treatment (Figure 1). The fresh weight reached its maximum on day 3 in four cultures and on day 4 in the remaining two cultures, followed by a decline thereafter (Figure 1d). In contrast, dry weight reached its maximum on day 3 in all cultures and gradually decreased thereafter (Figure 1e). The 72-h time point (day 3) was therefore selected for subsequent alkaloid and phytochemical analyses, while gene expression was evaluated at 24, 48, and 72 h after elicitation.

### 2.2. Effects of Fungal Elicitors and Methyl Jasmonate on Alkaloid Accumulation

The effects of RN3, methyl jasmonate (MeJA), and their combination on gravimetrically determined alkaloid content in *Catharanthus roseus* suspension cultures were evaluated 72 h after elicitor treatment (Figure 2a). Compared with the untreated control (9.24 mg g^−1^ DW), treatment with 1% RN3 significantly increased the gravimetrically determined alkaloid content to 11.71 mg g^−1^ DW, whereas 150 μM MeJA alone resulted in only a slight increase. Increasing the RN3 concentration to 2% reduced alkaloid accumulation compared with the 1% RN3 treatment. The highest gravimetrically determined alkaloid content (14.68 mg g^−1^ DW) was observed in cultures co-treated with 1% RN3 and 150 μM MeJA, representing a 58.9% increase over the untreated control (Figure 2a).

The TLC profiles showed differences in alkaloid band intensity among treatments (Figure 2b). Following visualization with Dragendorff’s reagent, differences in the intensity of alkaloid bands were observed among treatments, with the strongest bands detected in cultures co-treated with 1% RN3 and 150 μM MeJA. Together, these results indicated that co-treatment with 1% RN3 and 150 μM MeJA resulted in the highest alkaloid response among the treatments tested.

### 2.3. HPLC Analysis of Representative TIAs

HPLC (high-performance liquid chromatography) analysis of the alkaloid-enriched fractions was performed qualitatively to detect representative alkaloids by comparison with authentic standards. Distinct peaks were observed only in cultures treated with 1% RN3 or co-treated with 1% RN3 and 150 μM MeJA (Figure 3). No corresponding peaks were observed in the untreated control, MeJA-treated cultures, or cultures treated with 2% RN3. The chromatogram of the 1% RN3 treatment showed a major peak at 31.050 min (Figure 3b), whereas cultures co-treated with 1% RN3 and 150 μM MeJA exhibited two major peaks at 23.049 min and 31.050 min (Figure 3a). Comparison of the retention times with authentic catharanthine and vindoline standards indicated that the peaks at 31.050 and 23.049 min were consistent with catharanthine and vindoline, respectively (Figure 3c,d). Under the chromatographic conditions employed, no peaks corresponding to vinblastine or vincristine were detected in any of the samples analyzed.

### 2.4. Selective Transcriptional Responses of Representative TIA Pathway Genes

Based on the results of alkaloid accumulation, three elicitor treatments (1% RN3, 150 μM MeJA, and their combination) were selected for subsequent gene expression analysis. The expression levels of representative genes involved in the TIA biosynthetic pathway were determined by quantitative real-time PCR (qRT-PCR) at 24 h, 48 h, and 72 h after elicitation. The analyzed genes included the transcription factor genes *ORCA2* and *ORCA3* and structural genes representing different biosynthetic steps, namely *TDC*, *STR*, *G10H*, *SGD*, *T16H*, *T3O*, *T3R*, *D4H*, *DAT*, and *PRX1*. Relative gene expression was normalized using *RPS9* as the reference gene.

Among the examined genes, *ORCA2* and *ORCA3* exhibited markedly higher transcript levels in elicitor-treated cultures than in the untreated control. The greatest induction was observed after combined fungal elicitor and MeJA treatment, with *ORCA2* and *ORCA3* transcript levels increasing 29.8- and 18.8-fold, respectively, relative to the control at 24 h (Figure 4a,b). Although the transcript levels of *ORCA2* and *ORCA3* decreased at 48 and 72 h relative to their levels at 24 h, they remained significantly higher than those in the untreated control and other elicitor treatments at both time points. In contrast, no significant changes in transcript levels were detected in any of the tested structural genes at the time points examined (Supplementary Figure S10). Overall, the elicitation treatments markedly increased *ORCA2* and *ORCA3* transcript levels during the 24–72 h period examined, whereas no significant changes were detected in the tested structural genes at the sampled time points.

### 2.5. Isolation and Identification of Compounds from the H2L Fraction

To further characterize the metabolites present in the co-elicited culture, the culture was scaled up for compound isolation. The large-scale extraction yielded 6.8 g of the alkaloid-enriched H2L fraction. Repeated chromatographic separation of H2L afforded five compounds (Figure S1). The known compounds were identified as (+)-syringaresinol-β-D-glucoside (**2**) [22], tryptoline (**3**) [23], β-carboline (**4**) [24], and L-thymidine (**5**) [25] by comparison of their HR-ESI-MS and 1D and 2D NMR (nuclear magnetic resonance) spectroscopic data with published data.

Compound **1** was obtained as a pale-yellow wax and identified as a previously undescribed lignan glycoside, which was named catharalignan in this study. Its molecular formula was established as C_28_H_38_O_12_ based on HR-ESI-MS analysis, which showed a quasi-molecular ion peak at *m*/*z* 589.22565 [M + Na]^+^. The structure and absolute configuration of catharalignan (**1**) were determined by comprehensive spectroscopic analyses, including 1D and 2D NMR and circular dichroism (CD) experiments (Figure 5). The complete spectroscopic data of compound **1** are provided in Section 4.6 and Supplementary Materials (Figures S2–S9).

The structural elucidation of compound **1** is described below.

The ^1^H and ^13^C NMR spectroscopic data of **1** in CD_3_OD were indicative of a lignan derivative [26] with characteristic signals of two 1,3,4,5-tetrasubstituted aromatic rings [δ_C_ 140.5 (C-1), 105.3 (C-2 and C-6), 154.3 (C-3 and C-5), 135.8 (C-4)/δ_H_ 6.77 (2H, s, H-2 and H-6) and δ_C_ 136.4 (C-1′), 105.0 (C-2′ and C-6′), 149.3 (C-3′ and C-5′), 133.8 (C-4′)/δ_H_ 6.72 (2H, s, H-2′ and H-6′)], two oxymethines [δ_C_ 89.7 (C-7) and 90.4 (C-7′)/δ_H_ 4.70 (1H, d, J = 8.5 Hz, H-7) and 4.68 (1H, d, J = 8.5 Hz, H-7′)], two methines [δ_C_ 52.1 (C-8) and 52.2 (C-8′)/δ_H_ 1.83 (2H, m, H-8 and H-8′)], and two methyls [δ_C_ 14.0 (C-9) and 13.9 (C-9′)/δ_H_ 1.10 (H-9) and 1.06 (H-9′), each 3H, d, J = 5.5 Hz)]. Four methoxy groups [δ_C_ 57.1 (3-OMe and 5-OMe) and 56.8 (3′-OMe and 5′-OMe)/δ_H_ 3.90 (6H, s, 3-OMe and 5-OMe) and 3.89 (3′-OMe and 5′-OMe)] and one β-D-glucopyranose moiety [δ_C_ 105.5 (C-1″), 75.7 (C-2″), 77.8 (C-3″), 71.3 (C-4″), 78.3 (C-5″), and 62.6 (C-6″)] [27] were also observed. The β-D-glucopyranose moiety was confirmed by the ^1^H and ^13^C NMR data in CDCl_3_ at δ_C_ 106.4 (C-1″), 74.2 (C-2″), 76.7 (C-3″), 70.4 (C-4″), 76.0 (C-5″), and 62.5 (C-6″)/δ_H_ 4.57 (1H, d, J = 7.2 Hz, H-1″), 3.68 (1H, dd, J = 9.0, 7.2 Hz, H-2″), 3.61 (1H, t, J = 9.0 Hz, H-3″), 3.64 (1H, t, J = 9.0 Hz, H-4″), 3.38 (1H, m, H-5″), 3.81 (1H, dd, J = 12.0, 5.4 Hz, H_a_-6″), and 3.92 (1H, m, H_b_-6″). The NMR spectroscopic data of **1** resembled those of foliachinenoside C [27], except for the presence of two methyls in **1** instead of two oxymethylenes in foliachinenoside C.

In addition, the COSY connectivities of H-7/H-8/H-9 and H-7′/H-8′/H-9′ as well as HMBC correlations of H-2 with C-1, C-3, C-4, C-6, C-7; H-9 with C-8′, 3-OMe with C-3; H-2′ with C-1′, C-3′, C-4′, C-6′, C-7′; H-9′ with C-8, 3′-OMe with C-3′; and H-1″ with C-4 (Figure 5) clearly confirm the planar structure of **1**. The coupling constant *J* = 8.5/9.0 Hz of H-7 and H-7′ together with the chemical shifts of H-7, H-7′, H-8 and H-8′ indicated the *trans* relationship between each methyl group and the vicinal aryl group, as well as between H-8 and H-8′ [26]. Moreover, the relative configuration of **1** was supported by NOESY correlations between the following proton pairs (Figure 5): 2,6-H and 7-H, 8-H, 3,5-OMe; 7-H and 9-H_3_, 8′-H; 8-H and 7′-H; 2′,6′-H and 7′-H, 8′-H, 3′,5′-OMe; 7′-H and 9′-H_3_ [24]. Finally, the absolute configuration was assigned as 7*S*, 7′*S*, 8*R*, 8′*R* based on the CD spectrum, which showed a negative Cotton effect at 243 nm [27,28,29].

## 3. Discussion

### 3.1. Combined Fungal Elicitor and MeJA Altered Cell Growth and Promoted Alkaloid Accumulation

In the present study, co-treatment with the RN3 fungal elicitor and MeJA significantly increased total alkaloid accumulation while moderately reducing biomass compared with the untreated control. Among all treatments, the combined treatment produced the highest total alkaloid accumulation. This result suggests that the combination of RN3 and MeJA was more effective than either elicitor alone under the tested conditions.

Reduced biomass following elicitor treatment has been reported in *C. roseus* suspension cultures. Zhao et al. [30] found that fungal elicitors inhibited cell growth to different degrees while increasing indole alkaloid accumulation. Similar effects have been observed in *C. roseus* cultures treated with other elicitors, including MeJA and chitin [31]. Elicitation can activate defense responses and stimulate specialized metabolite production, which may occur together with reduced cell growth [32,33].

The higher alkaloid accumulation observed under co-elicitation may reflect complementary effects of RN3 and MeJA. Fungal elicitors and MeJA can engage distinct but overlapping defense signaling pathways and stimulate specialized metabolism [32,33,34]. Similar responses have been reported in *C. roseus* cultures treated with combined elicitors, although the effect depended on the elicitor combination [31]. Zhao et al. [35] demonstrated that selected combinations of fungal and chemical elicitors exerted synergistic effects on alkaloid accumulation, with different elicitor combinations preferentially enhancing catharanthine and ajmalicine production. A similar enhancement of specialized metabolite accumulation following co-elicitation has been reported in *Lavandula angustifolia* suspension cultures. Yazdi et al. [36] found that combined treatment with 50 µM MeJA and 1 g/L yeast extract increased intracellular rosmarinic acid accumulation to approximately 17.1 mg/g DW after 24 h, markedly exceeding the levels obtained with either elicitor alone. However, prolonged co-treatment to 72 h resulted in a substantial decline in rosmarinic acid accumulation, indicating that the response to combined elicitation was strongly dependent on treatment duration.

The timing and duration of elicitor treatment can also affect alkaloid accumulation. Zhao et al. [30] reported that the culture age and duration of fungal elicitation influenced alkaloid production in *C. roseus*. The authors found that fungal elicitors were most effective when added during the log phase, with three to four days of treatment giving high alkaloid production. In the present study, the RN3 fungal elicitor and MeJA were applied on day 18, during the late exponential phase of H01 cell growth, and cells were harvested after three days of elicitation. This treatment period may have contributed to the increased alkaloid accumulation observed in the present study.

Accordingly, the co-treatment with RN3 and MeJA was selected for subsequent large-scale phytochemical investigation, while selected elicitor treatments were further evaluated by HPLC and gene expression analysis.

### 3.2. Targeted Analysis of Representative TIAs

HPLC analysis showed peaks corresponding to representative TIAs based on comparison with authentic standards only in cultures treated with 1% RN3 or co-treated with 1% RN3 and 150 μM MeJA. Catharanthine was detected after RN3 treatment alone, whereas both catharanthine and vindoline were detected after co-treatment with RN3 and MeJA. Neither catharanthine nor vindoline was detected in the untreated control, MeJA-treated cultures, or cultures treated with 2% RN3 under the chromatographic conditions used. Thus, the combined treatment was associated with both increased gravimetrically determined alkaloid content and a change in the pattern of detectable alkaloids.

The absence of detectable vinblastine and vincristine in the present study is consistent with previous observations in *C. roseus* suspension cultures. Zavala-Ortiz et al. [37] reported that the CRBY-1 suspension culture accumulated ajmalicine and catharanthine, whereas vinblastine and vincristine were not detected. The authors further suggested that the absence of vincristine production in the suspension culture may be associated with limited *D4H* expression. A similar detection pattern was observed in the H01 suspension culture, in which catharanthine and vindoline were detected after co-treatment, whereas vinblastine and vincristine remained undetected under the conditions used. Vinblastine and vincristine are downstream bisindole alkaloids formed through the coupling of catharanthine and vindoline [13]. In the present study, their absence despite the detection of both monomeric alkaloids may indicate limited conversion to downstream bisindole alkaloids under the conditions used.

The increase in gravimetrically determined alkaloid content may also reflect contributions from other alkaloid constituents not examined by the targeted HPLC analysis. The HPLC analysis was limited to selected representative TIAs and was qualitative rather than quantitative. The isolation of tryptoline and β-carboline from the co-elicited culture further supports the presence of other alkaloid constituents not included in the targeted HPLC analysis.

### 3.3. Gene Expression Under Combined Fungal Elicitor and MeJA Elicitation

Co-elicitation with RN3 and MeJA strongly induced the transcription of *ORCA2* and *ORCA3*, with 29.8- and 18.8-fold increases, respectively, relative to the untreated control. ORCA2 and ORCA3 are jasmonate-responsive AP2/ERF transcription factors involved in the regulation of TIA biosynthesis [16,18,38]. Previous studies have shown that ORCA2 and ORCA3 regulate several genes in TIA biosynthesis, including *TDC* and *STR*, while ORCA3 also regulates the expression of downstream genes such as *D4H* [17,38,39].

Despite the strong induction of *ORCA2* and *ORCA3*, none of the structural genes examined in the present study showed significant transcriptional upregulation. This result indicates that induction of the regulatory factors was not accompanied by a coordinated increase in the transcript levels of the structural genes examined. Previous studies have likewise shown that TIA biosynthetic genes do not respond uniformly to regulatory perturbation. *ORCA3* overexpression increased the expression of several TIA biosynthetic genes, including *TDC*, *STR*, and *D4H*, whereas other genes showed little or no response [39]. In particular, *SGD* expression decreased following *ORCA3* overexpression [40]. Sun and Peebles [40] further showed that co-overexpression of *ORCA3* and *SGD*, rather than *ORCA3* overexpression alone, resulted in a clear increase in TIA accumulation. These findings indicate that induction of *ORCA3* alone is not sufficient to ensure coordinated activation of all downstream TIA biosynthetic genes.

The contrasting expression patterns may also reflect differences in the temporal kinetics of the elicitation response. In *C. roseus* suspension cells, fungal elicitation induces a rapid jasmonate response, with jasmonic acid accumulation occurring within 1 h and peaking at 3 h, followed by induction of *Tdc* and *Str* transcripts within the first several hours after elicitation [41]. Thus, transient induction of some structural genes before the first sampling point at 24 h cannot be excluded in the present study.

Differential responses of TIA biosynthetic genes have also been reported following fungal elicitation. Pandey et al. [14] found that fungal endophytes increased the expression of several TIA biosynthetic genes, whereas other genes showed little change or were downregulated. The responses of regulatory genes also differed between fungal treatments. *ORCA3* was induced by one of the fungal endophytes, whereas *ORCA2* showed little change. In addition, the expression of the negative regulators ZCT1–3 and GBFs was also altered [14]. These findings support the view that TIA biosynthesis is regulated by a network of positive and negative transcriptional regulators rather than by a single transcription factor [42]. Thus, the strong induction of *ORCA2* and *ORCA3* observed in the present study may not have been sufficient to induce all structural genes examined.

In addition to transcriptional regulation, ORCA activity may be regulated beyond transcript abundance. Paul et al. [18] demonstrated that CrMAPK3-mediated phosphorylation enhanced the transactivation activity of ORCA3, indicating that the abundance of *ORCA3* transcripts does not necessarily directly reflect the transcriptional activity of the corresponding protein. Individual ORCA factors also show overlapping but distinct effects on downstream TIA genes [18]. Therefore, the strong induction of *ORCA2* and *ORCA3* observed here does not necessarily imply simultaneous activation of all structural genes at the sampled time points. A similar discrepancy was observed under MeJA treatment alone, where *ORCA2* and *ORCA3* were upregulated without a corresponding increase in gravimetrically determined alkaloid content. This finding further suggests that induction of these transcriptional regulators alone may be insufficient to drive coordinated activation of the downstream TIA biosynthetic pathway. The transcriptional response to MeJA may precede subsequent metabolic changes, while the lack of significant changes in several downstream structural genes may partly explain the absence of a corresponding increase in alkaloid content.

In the present study, catharanthine and vindoline were nevertheless detected after RN3 and MeJA co-treatment. This observation suggests that transcript levels at the sampling times did not directly reflect the detectable presence of these alkaloids. Pandey et al. [14] similarly found no direct relationship between *PRX1* expression and vindoline content in their study. Tang et al. [15] also reported that changes in the expression of TIA biosynthetic genes did not always correspond to changes in vindoline and catharanthine accumulation following elicitation. Therefore, the accumulation of TIA metabolites may not be directly predicted from the transcript levels of individual pathway genes at selected time points.

Taken together, these findings suggest that RN3 and MeJA co-treatment elicited a strong transcriptional response of *ORCA2* and *ORCA3*, but this response was not accompanied by coordinated induction of the structural genes examined. The observed alkaloid accumulation may therefore reflect the cumulative response to elicitation, including transcriptional events occurring before 24 h and regulatory processes not directly captured by transcript abundance at the sampled time points. The detection of catharanthine and vindoline under the co-treatment further indicates that transcript abundance at the measured time points did not directly predict the detectable TIA profile.

### 3.4. Tryptamine-Derived Alkaloids in Elicited H01 Suspension Cultures

The isolation of β-carboline (2 mg) and tryptoline (22 mg) from the alkaloid-enriched fraction indicates that tryptamine-derived alkaloids were present in the co-elicited H01 suspension cultures. Both compounds are β-carboline alkaloids derived from tryptamine through alternative Pictet–Spengler-related reactions and are distinct from the canonical TIA pathway [43,44,45].

In the canonical TIA pathway, tryptophan is converted to tryptamine by tryptophan decarboxylase (TDC). Tryptamine then condenses with secologanin through strictosidine synthase (STR) to form strictosidine, the central precursor of TIA biosynthesis [3,46]. The detection of β-carboline and tryptoline together with catharanthine and vindoline indicates the coexistence of tryptamine-derived alkaloids and representative TIAs in the co-elicited H01 cultures. This observation suggests that tryptamine-derived metabolism may proceed through routes beyond the canonical TIA pathway. Consistent with this observation, previous studies have reported that fungal elicitation of *C. roseus* suspension cultures induced the accumulation of tryptamine together with several structurally distinct indole alkaloids, including N-acetyltryptamine, strictosidine lactam, ajmalicine, tabersonine, lochnericine, and catharanthine [47].

### 3.5. Isolation of a New Lignan from H01 Suspension Cultures

A notable finding of the present study was the isolation of a previously undescribed lignan from the co-elicited *C. roseus* suspension cultures. To the best of our knowledge, this compound has not been previously reported from natural or cultured sources.

Although *C. roseus* cell cultures have been extensively studied for terpenoid indole alkaloid production, reports on the isolation and structural characterization of individual lignans from suspension cultures remain limited. Plant suspension cultures have been used for the production and investigation of lignans and other specialized metabolites [48,49,50]. In vitro cultures can also produce metabolite profiles that differ from those of the intact plant. A relevant example is *Phyllanthus niruri*, in which Elfahmi et al. [51] isolated cubebin dimethyl ether from suspension cultures although this lignan had not previously been detected in the intact plant. These findings highlight the potential of suspension cultures to accumulate metabolites that may be less readily detected in the source plant.

Elicitation can further modify lignan accumulation and composition. In *Linum* suspension cultures, methyl jasmonate and fungal elicitors have been reported to enhance lignan biosynthesis, alter the accumulation of monolignol-derived compounds, and modulate the expression of genes involved in lignan biosynthesis [52,53,54]. These studies indicate that elicitation can influence lignan biosynthesis and the accumulation of related phenylpropanoid-derived metabolites. In the present study, however, catharalignan was identified only from the co-elicited culture, and its accumulation was not quantitatively compared among the different elicitation treatments. The isolation of the previously undescribed lignan therefore expands the range of specialized metabolites identified from the *C. roseus* suspension culture system, but does not establish that RN3 and MeJA induced its biosynthesis. Whether these elicitors influence lignan biosynthesis in H01 cells remains to be determined.

## 4. Materials and Methods

### 4.1. Plant Material and Suspension Cell Culture

The *Catharanthus roseus* (L.) G. Don suspension cell line H01 was established from stem-derived calli of in vitro-grown plants in our laboratory in 2023. The cell line originated from *C. roseus* var. *roseus* collected from Lang Co, Hue City, Vietnam.

Suspension cultures were maintained in Murashige and Skoog (MS) basal medium (M0221, Duchefa Biochemie, Haarlem, The Netherlands) supplemented with MS vitamin mixture (M0409, Duchefa Biochemie, Haarlem, The Netherlands), 30 g L^−1^ sucrose, 1.0 mg L^−1^ kinetin (Duchefa Biochemie, Haarlem, The Netherlands), and 1.0 mg L^−1^ α-naphthaleneacetic acid (NAA; Duchefa Biochemie, Haarlem, The Netherlands). The pH was adjusted to 5.8 before autoclaving. Suspension cell cultures were grown in 250-mL Bomex Erlenmeyer flasks containing 100 mL of medium and inoculated with 10 mL of suspension culture. The cultures were incubated in darkness at 25 °C and 120 rpm in a shaker incubator. Subcultures were performed every 21 days.

Cell morphology was examined using an inverted microscope (CKX53, Evident Scientific, Tokyo, Japan) at 50× magnification (5× objective and 10× eyepiece). For morphological observation, a 500 μL aliquot of the cell suspension was spread on an adhesive cell-culture Petri dish and observed under the inverted microscope without staining.

#### Determination of Cell Fresh and Dry Weights

Cell biomass was collected daily for six consecutive days after elicitor treatment. At each sampling time, the entire 100 mL culture from a separate culture flask was collected by filtration through a funnel fitted with Whatman filter paper. The cells were washed three times with distilled water, and excess water was removed by gently blotting the cells with dry filter paper before weighing to determine the fresh weight (FW). The cells were then dried at 55 °C until a constant weight was reached and weighed to determine the dry weight (DW).

### 4.2. Preparation and Application of Elicitors

#### 4.2.1. Preparation of RN3 Fungal Elicitor

The RN3 strain of *Chaetomium funicola* Cooke was previously isolated from *C. roseus* root samples as described by Linh et al. [12] and maintained at −80 °C. The fungus was activated on Hansen agar medium at 30 °C for 3 days and subsequently cultured in 100 mL of Hansen liquid medium in 250-mL Erlenmeyer flasks at 30 °C and 180 rpm for 7 days. The mycelia were collected by filtration, washed with sterile distilled water, dried at 65 °C to a constant weight, and ground into a fine powder. Dried mycelia (10 g) were suspended in 100 mL of distilled water, autoclaved at 120 °C for 15 min, and centrifuged at 4500 rpm (3305× *g*) for 15 min, following the procedure described by Baldi et al. [55] and previously applied to *C. roseus* suspension cultures by our group [12]. The resulting supernatant was used as the stock solution of the RN3 fungal cell extract.

#### 4.2.2. Elicitor Treatments

The concentrations of RN3 fungal elicitor (1% and 2%, *v*/*v*) were selected based on our previous study [12], while the MeJA concentrations of 100 and 150 μM were selected based on previously reported effective concentrations used in *C. roseus* cell culture systems [56,57]. RN3 fungal elicitor stock solution was added to the cultures to obtain final concentrations of 1% or 2% (*v*/*v*). Methyl jasmonate (MeJA, 95%; Sigma-Aldrich, St. Louis, MO, USA; product no. 392707) was added directly to the cell suspension without prior dissolution in an additional solvent. For 100 mL of cell suspension, 2.29 or 3.44 μL of MeJA was added to obtain final concentrations of 100 or 150 μM, respectively. All elicitor treatments were applied on day 18 of culture. Treatments consisted of 1% or 2% (*v*/*v*) RN3 fungal elicitor, 100 or 150 μM MeJA, or a combination of 1% (*v*/*v*) RN3 fungal elicitor and 150 μM MeJA. Control cultures received an equal volume of sterile distilled water. For biomass determination, cells were harvested daily for six consecutive days after elicitor treatment. For gene expression analysis, cells were harvested at 24, 48, and 72 h after elicitor treatment. The 24-, 48-, and 72-h sampling points were selected to monitor gene expression throughout the three-day elicitation period and to relate transcriptional responses to the subsequent alkaloid response at 72 h. Samples were immediately frozen in liquid nitrogen and stored at −80 °C until RNA extraction.

### 4.3. Gravimetric Determination of Alkaloid Content

For gravimetric determination of alkaloid content , cells were harvested 72 h after elicitor treatment. Alkaloid content was determined gravimetrically using a method adapted from Gomaa et al. [58], with the following modifications. Suspension cells were harvested by filtration, washed with distilled water, and dried at 55 °C to a constant weight. The dried biomass was extracted repeatedly with methanol, and the combined extracts were concentrated under reduced pressure at 55 °C to obtain the crude methanolic extract. The residue was suspended in distilled water, acidified with concentrated HCl, and extracted with ethyl acetate to remove neutral compounds. The aqueous phase was adjusted to pH 8 with 1 M NH_4_OH and extracted three times with ethyl acetate. The combined organic phases were concentrated under reduced pressure at 55 °C to obtain the alkaloid-enriched fraction. The dried alkaloid-enriched fraction was weighed, and the gravimetrically determined alkaloid content was expressed as mg g^−1^ DW. Three independent biological replicates were analyzed for each treatment.

For thin-layer chromatography (TLC) analysis, the alkaloid-enriched fraction was dissolved in methanol, and 10 μL of the solution was applied to silica gel 60 F_254_ plates (1.05554.0001, Merck, Darmstadt, Germany). The plates were developed using dichloromethane/methanol 10:1, *v*/*v*) as the mobile phase and subsequently sprayed with Dragendorff’s reagent for alkaloid visualization.

### 4.4. HPLC Analysis

HPLC analysis was performed qualitatively to detect representative alkaloids by comparison of retention times and UV absorption spectra with authentic standards. The alkaloid-enriched fraction obtained in Section 4.3 was dissolved in methanol at a concentration of 10 mg mL^−1^ for HPLC analysis. Chromatographic analysis was performed using an Agilent 1200 Series HPLC system (Agilent Technologies, Santa Clara, CA, USA) equipped with a diode-array detector (DAD) and an Agilent Eclipse XDB-C18 column (4.6 × 250 mm, 5 μm). The mobile phase consisted of acetonitrile and 25 mM ammonium acetate in water (50:50, *v*/*v*). Separation was performed at a flow rate of 0.5 mL min^−1^ for 40 min with the column maintained at 25 °C. The injection volume was 10 μL, and chromatograms were recorded over the wavelength range of 210–365 nm. Catharanthine (Sigma-Aldrich, St. Louis, MO, USA), vindoline (Acros Organics, Geel, Belgium), vinblastine sulfate (Sigma-Aldrich, St. Louis, MO, USA), and vincristine sulfate (Sigma-Aldrich, St. Louis, MO, USA) were used as reference standards at a concentration of 1 mg mL^−1^.

### 4.5. RNA Extraction and RT-qPCR Analysis

Total RNA was extracted from suspension cells collected 24, 48, and 72 h after elicitor treatment using a modified TRIzol-based method (TRIzol Reagent, Ambion, Thermo Fisher Scientific, Austin, TX, USA). RNA integrity was verified by 0.8% agarose gel electrophoresis, and RNA concentration and purity were determined using a NanoDrop™ 2000c spectrophotometer (Thermo Fisher Scientific, Waltham, MA, USA). Genomic DNA was removed using DNase I (Thermo Fisher Scientific, Vilnius, Lithuania), and 1 μg of total RNA was reverse transcribed into cDNA using the RevertAid First Strand cDNA Synthesis Kit (Thermo Fisher Scientific, Vilnius, Lithuania) according to the manufacturer’s instructions.

Expression levels of genes associated with the terpenoid indole alkaloid (TIA) biosynthetic pathway, including *TDC*, *STR*, *G10H*, *SGD*, *T16H*, *T3O*, *T3R*, *D4H*, *DAT*, *ORCA2*, *ORCA3*, and *PRX1*, were quantified by RT-qPCR using FastStart Essential DNA Green Master (Roche Diagnostics, Mannheim, Germany) on a QuantStudio™ 5 Real-Time PCR System (Applied Biosystems™, Thermo Fisher Scientific, Waltham, MA, USA). Primer sequences are listed in Table S1, and *RPS9* was used as the reference gene. Relative gene expression was calculated using the 2^−ΔΔCt^ method [59]. For gene expression analysis, three independent biological replicates were analyzed for each selected treatment, and expression levels were calculated relative to the untreated control, which was set to 1.

### 4.6. Compound Isolation

#### 4.6.1. Cell Suspension Preparation

For large-scale extraction, cell suspension cultures were grown in 50 × 2-L Erlenmeyer flasks, each containing 1 L of suspension culture, and treated with 1% RN3 fungal elicitor and 150 μM MeJA, as described in Section 4.2. Cells were harvested after 72 h of treatment, and the biomass from all 50 flasks was pooled before extraction according to the procedure described in Section 4.3 to obtain the H2L fraction.

#### 4.6.2. General Experimental Isolation Procedures

Optical rotations were measured on a JASCO P-2000 polarimeter (Tokyo, Japan). Circular dichroism (CD) spectra were recorded on a Chirascan spectropolarimeter (Applied Photophysics, Leatherhead, UK). High-resolution mass spectra (HRMS) were acquired on an Orbitrap Exploris 120 system (Thermo Fisher Scientific, Waltham, MA, USA). The ^1^H NMR (nuclear magnetic resonance) and ^13^C NMR spectra were recorded on AVANCE III HD 500 and AVANCE NEO 600 FT-NMR spectrometers (Bruker, Karlsruhe, Germany) using TMS as an internal standard. High-performance liquid chromatography (HPLC) was performed on an Agilent 1260 Infinity II system (Agilent Technologies, Santa Clara, CA, USA) equipped with a G7112B Binary Pump, a G7115A diode array detector, and a G7129A auto-sampler. Column chromatography (CC) was carried out using Diaion HP-20 (Supelco, Bellefonte, PA, USA), silica gel (Kieselgel 60, 70–230 mesh and 230–400 mesh, Merck, Darmstadt, Germany), and reversed-phase silica gel (ODS-A, 12 nm S-150 µm, YMC, Kyoto, Japan). Pre-coated silica gel 60 F_254_ (1.05554.0001, Merck, Darmstadt, Germany) and RP-18 F_254S_ plates (1.15389.0001, Merck) were used for TLC, and spots were visualized by spraying with 10% aqueous H_2_SO_4_, followed by heating for 3–5 min.

#### 4.6.3. Isolation of Compounds from the H2L Alkaloid-Enriched Fraction

The H2L residue (6.8 g) was subjected to silica gel CC and eluted stepwise with CH_2_Cl_2_−MeOH (20:1, 10:1, 5:1, 2:1, and 0:1, *v*/*v*) to afford 12 fractions (A1–A12). Fraction A11 (700 mg) was further separated by Sephadex LH-20 CC using MeOH–H_2_O (3:1, *v*/*v*), followed by silica gel CC with EtOAc–MeOH (3:1, *v*/*v*) and HPLC (Cosmosil 5C18-MS-II column, 4.6 × 250 mm, 5 μm; flow rate: 1.0 mL/min; ACN–H_2_O, 80:20) to yield compound **3** (22 mg). Compounds **1** (2.5 mg), **2** (12 mg), and **5** (2.0 mg) were isolated from fraction A9 (700 mg) by HPLC (Cosmosil 5C18-MS-II column, 4.6 × 250 mm, 5 μm; flow rate: 1.0 mL/min; gradient elution with ACN–H_2_O from 5:95 to 40:60 over 30 min). Fractions A4 and A5 were combined (40 mg) and further purified by reversed-phase silica gel CC using acetone–H_2_O (2:1, *v*/*v*), followed by HPLC (Cosmosil 5C18-MS-II column, 4.6 × 250 mm, 5 μm; flow rate: 1.0 mL/min; ACN–H_2_O, 45:55) to afford compound **4** (2.0 mg).

Catharalignan (**1**): Pale yellow wax; [α]_D_^20^ −8.5 (*c* 0.05, MeOH); CD (MeOH) *λ*_max_ (mdeg): 243 (−0.76) nm; ^1^H NMR (500 MHz, CD_3_OD): *δ*_H_ 6.77 (2H, s, H-2 and H-6), 4.70 (1H, d, *J* = 8.5 Hz, H-7), 1.83 (2H, m, H-8 and H-8′), 1.10 (3H, d, *J* = 5.5 Hz, H-9), 3.90 (6H, s, 3-OMe and 5-OMe), 6.72 (2H, s, H-2′ and H-6′), 4.68 (1H, d, *J* = 8.5 Hz, H-7′), 1.06 (3H, d, *J* = 5.5 Hz, H-9′), 3.89 (6H, s, 3′-OMe and 5′-OMe), 4.88 (1H, H-1″), 3.50 (1H, H-2″), 3.44 (2H, H-3″ and H-4″), 3.24 (1H, m, H-5″), 3.70 (1H, dd, *J* = 11.5, 5.0 Hz, H_a_-6″), and 3.80 (1H, H_b_-6″) ppm; ^13^C NMR data (125 MHz, CD_3_OD): *δ*_C_ 140.5 (C-1), 105.3 (C-2 and C-6), 154.3 (C-3 and C-5), 135.8 (C-4), 89.7 (C-7), 52.1 (C-8), 14.0 (C-9), 57.1 (3-OMe and 5-OMe), 136.4 (C-1′), 105.0 (C-2′ and C-6′), 149.3 (C-3′ and C-5′), 133.8 (C-4′), 90.4 (C-7′), 52.2 (C-8′), 13.9 (C-9′), 56.8 (3′-OMe and 5′-OMe), 105.5 (C-1″), 75.7 (C-2″), 77.8 (C-3″), 71.3 (C-4″), 78.3 (C-5″), and 62.6 (C-6″) ppm; ^1^H NMR (600 MHz, CDCl_3_): *δ*_H_ 6.66 (2H, s, H-2 and H-6), 4.66 (1H, d, *J* = 9.0 Hz, H-7), 1.78 (2H, m, H-8 and H-8′), 1.10 (3H, d, *J* = 6.0 Hz, H-9), 3.90 (6H, s, 3-OMe and 5-OMe), 6.62 (2H, s, H-2′ and H-6′), 4.63 (1H, d, *J* = 9.0 Hz, H-7′), 1.06 (3H, d, *J* = 5.4 Hz, H-9′), 3.91 (6H, s, 3′-OMe and 5′-OMe), 4.57 (1H, d, *J* = 7.2 Hz, H-1″), 3.68 (1H, dd, *J* = 9.0, 7.2 Hz, H-2″), 3.61 (1H, t, *J* = 9.0 Hz, H-3″), 3.64 (1H, t, *J* = 9.0 Hz, H-4″), 3.38 (1H, m, H-5″), 3.81 (1H, dd, *J* = 12.0, 5.4 Hz, H_a_-6″), and 3.92 (1H, m, H_b_-6″); ^13^C NMR data (150 MHz, CDCl_3_): *δ*_C_ 140.0 (C-1), 103.1 (C-2 and C-6), 152.7 (C-3 and C-5), 134.7 (C-4), 88.2 (C-7), 51.1 (C-8), 14.1 (C-9), 56.4 (3-OMe and 5-OMe), 134.3 (C-1′), 102.9 (C-2′ and C-6′), 147.1 (C-3′ and C-5′), 133.1 (C-4′), 88.8 (C-7′), 51.0 (C-8′), 13.8 (C-9′), 56.4 (3′-OMe and 5′-OMe), 106.4 (C-1″), 74.2 (C-2″), 76.7 (C-3″), 70.4 (C-4″), 76.0 (C-5″), and 62.5 (C-6″); HR-ESI-MS *m*/*z* 589.22565 [M + Na]^+^ (calcd. for C_28_H_38_O_12_Na^+^, 589.22555).

### 4.7. Data Analysis

Data were obtained from three independent replicates per treatment and are presented as mean ± standard error. Differences were considered statistically significant at *p* < 0.05 and were evaluated using Duncan’s multiple range test in IBM SPSS Statistics, Version 20 (IBM Corp., Armonk, NY, USA).

## 5. Conclusions

The present study shows that co-elicitation with RN3 and methyl jasmonate enhanced total alkaloid accumulation in *Catharanthus roseus* H01 suspension cultures. The treatment markedly increased the transcript levels of *ORCA2* and *ORCA3*, whereas the structural genes examined showed no significant transcriptional induction. Catharanthine and vindoline were detected following co-elicitation, while two tryptamine-derived alkaloids and the previously undescribed lignan catharalignan were isolated from the co-elicited culture. These findings demonstrate that co-elicited H01 suspension cultures contain both representative TIAs and tryptamine-derived alkaloids, as well as the previously undescribed lignan catharalignan, highlighting the value of this culture system for investigating elicitor-associated alkaloid responses and for phytochemical discovery.

## Figures and Tables

**Figure 1:**
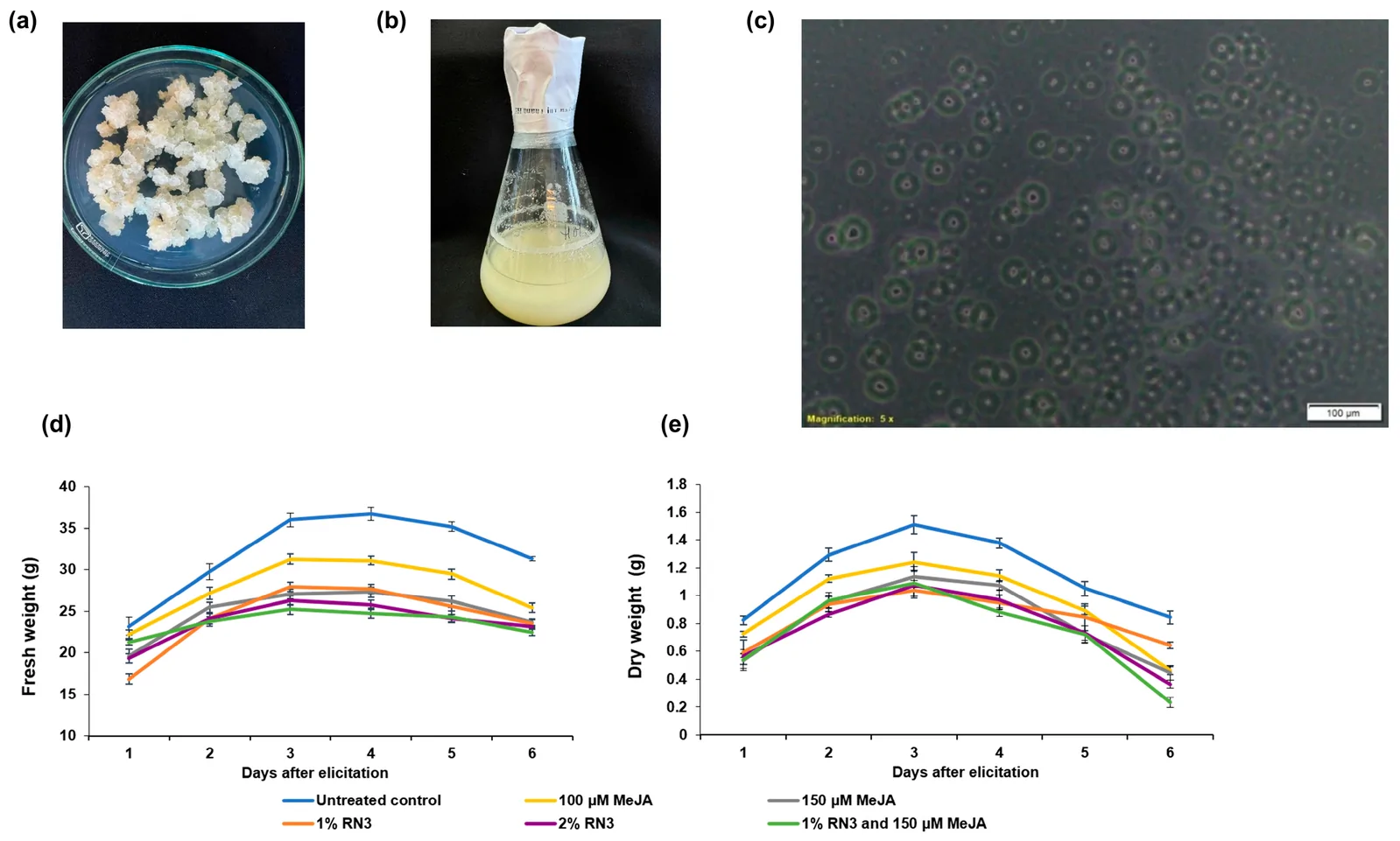
*Catharanthus roseus* H01 cell culture and growth kinetics under elicitation. (**a**) H01 cells maintained on solid medium in a 9-cm Petri dish. (**b**) H01 suspension culture on day 21 before harvest. (**c**) Microscopic morphology of H01 suspension cells. (**d**) Fresh weight (g per 100 mL culture) over six days after elicitor treatment. (**e**) Dry weight (g per 100 mL culture) over six days after elicitor treatment. At each sampling time, biomass was collected from three independent flasks. Data are presented as mean ± SE (*n* = 3).

**Figure 2:**
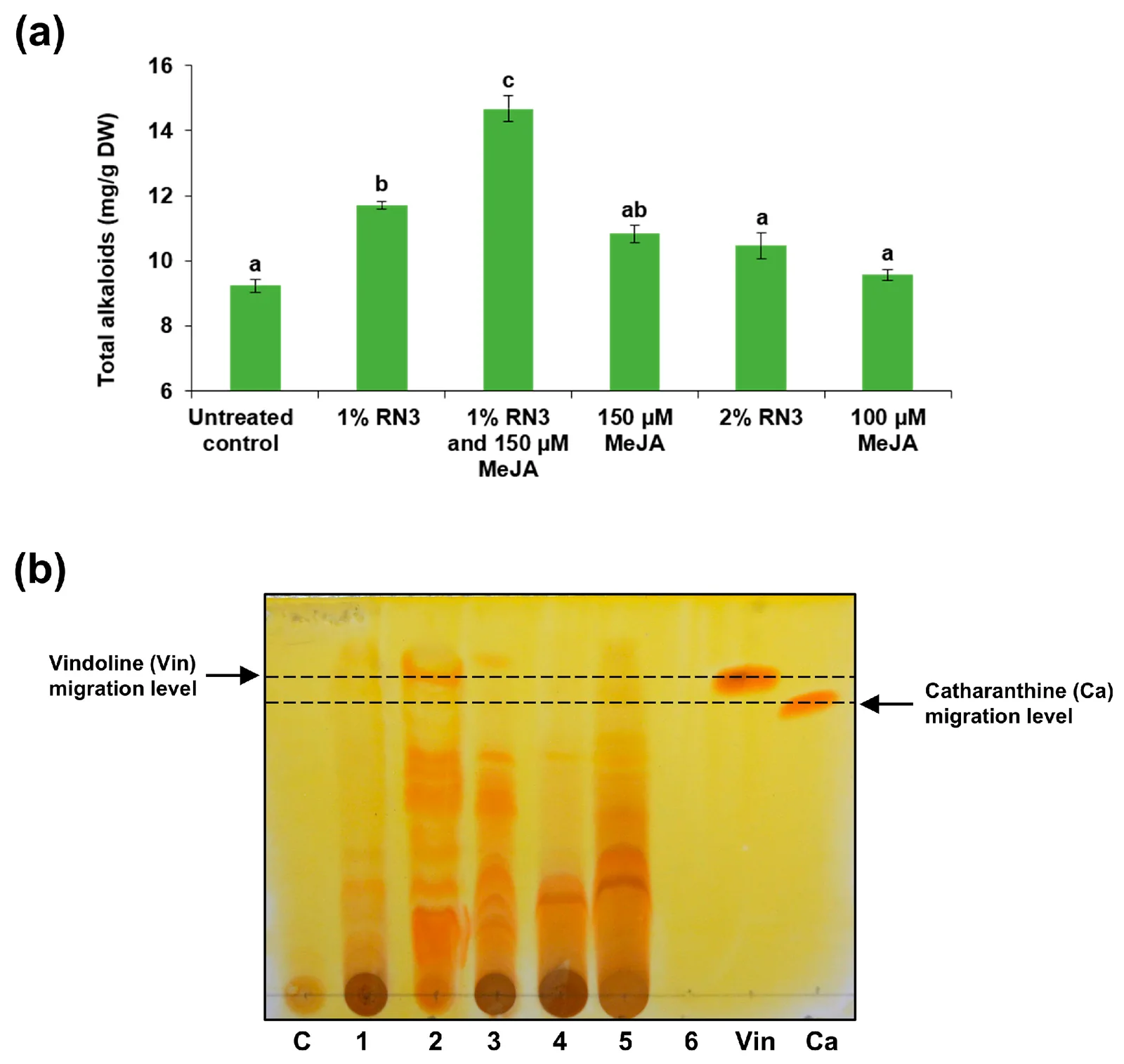
Effect of RN3 fungal elicitor and methyl jasmonate (MeJA) on alkaloid accumulation in *Catharanthus roseus* H01 suspension cultures 72 h after elicitor treatment. (**a**) Gravimetrically determined alkaloid content (mg/g DW) under different elicitor treatments. Different letters indicate significant differences among treatments (*p* < 0.05) (**b**) Thin-layer chromatographic profiles of alkaloid-enriched fractions from H01 suspension cultures subjected to different treatments, visualized with Dragendorff’s reagent. Lane C, untreated control; lane 1, 1% RN3; lane 2, 1% RN3 and 150 μM MeJA; lane 3, 150 μM MeJA; lane 4, 2% RN3; lane 5, 100 μM MeJA; lane B, solvent blank; Vin, authentic vindoline standard; Ca, authentic catharanthine standard. The horizontal lines indicate the migration levels of the corresponding authentic standards for comparison with the sample profiles. The TLC plates were developed with dichloromethane/methanol (10:1, *v*/*v*).

**Figure 3:**
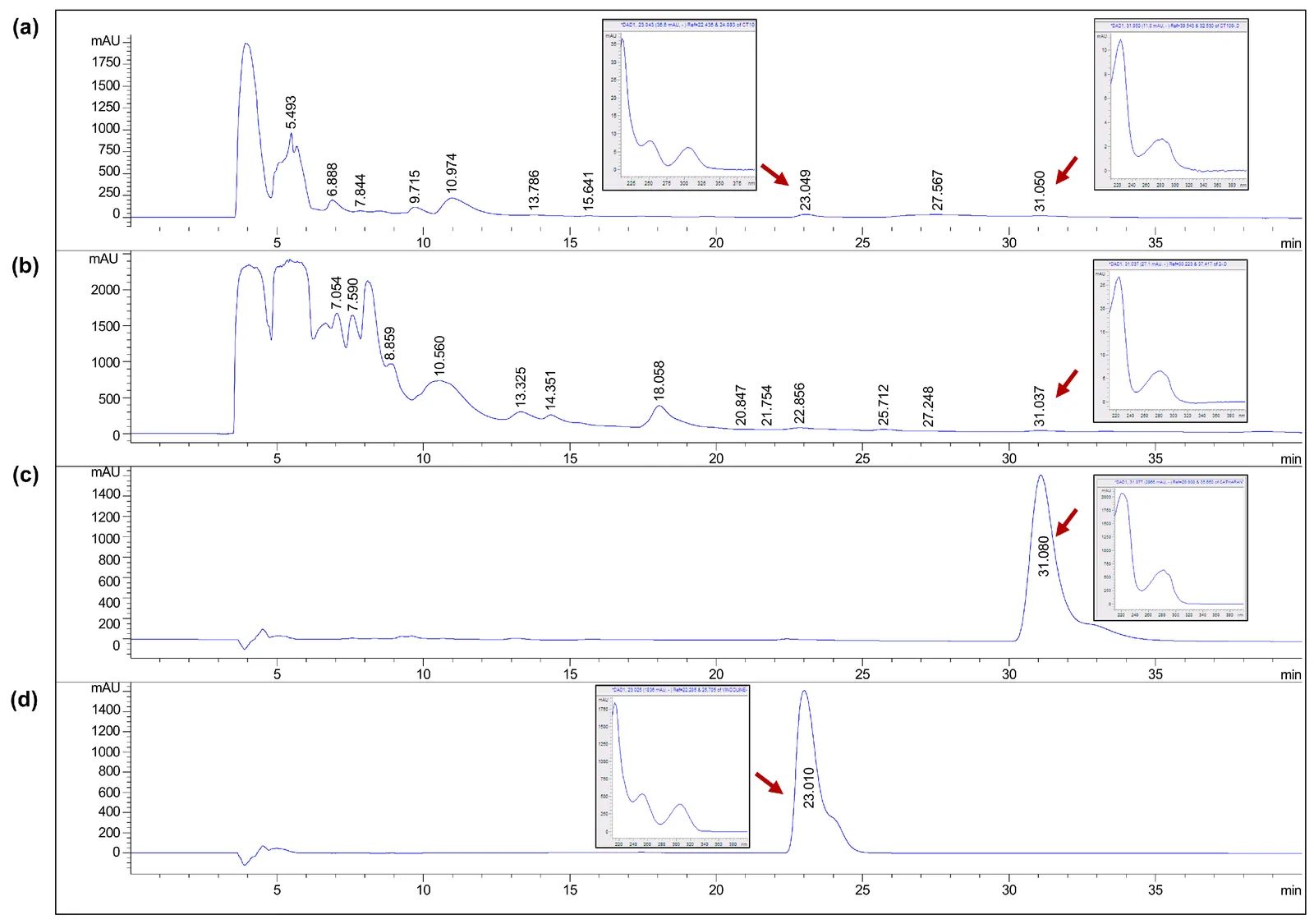
HPLC chromatograms of representative alkaloid-enriched fractions from *Catharanthus roseus* H01 suspension cultures and authentic standards. (**a**) Alkaloid-enriched fraction from cultures co-treated with 1% RN3 fungal elicitor and 150 μM methyl jasmonate (MeJA). (**b**) Alkaloid-enriched fraction from cultures treated with 1% RN3 fungal elicitor. (**c**) Catharanthine standard. (**d**) Vindoline standard.

**Figure 4:**
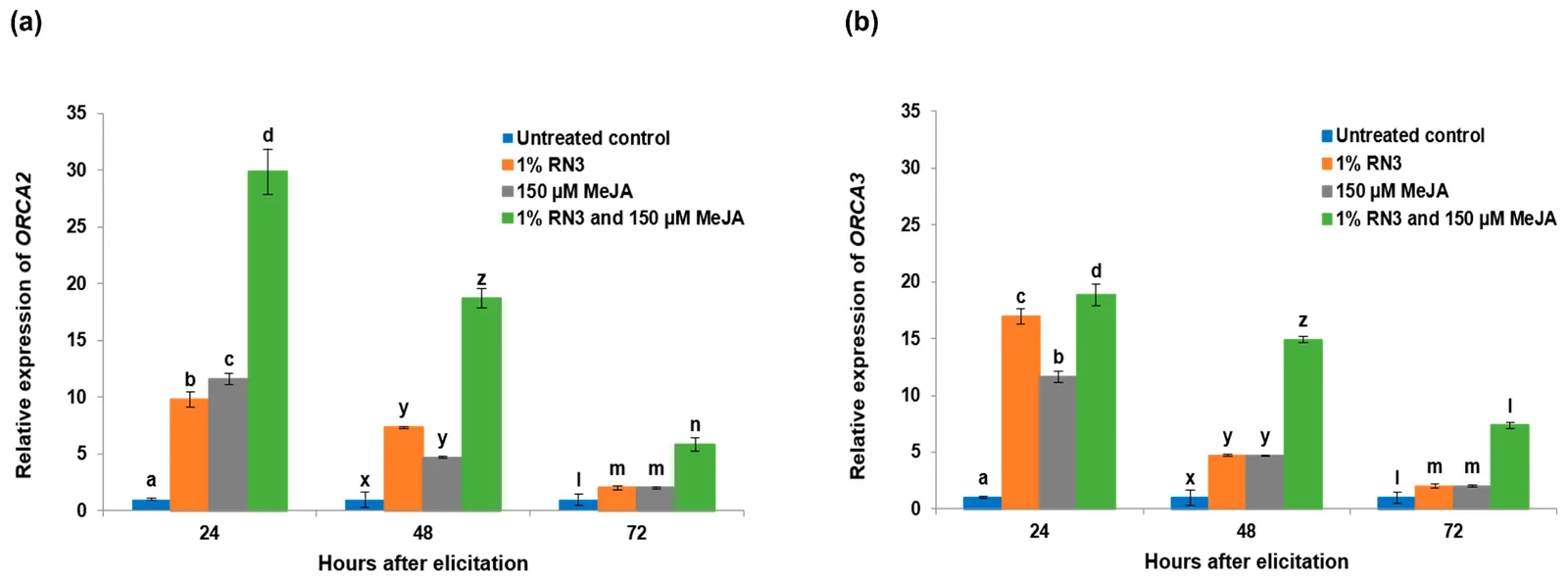
Expression of the transcription factor genes ORCA2 (**a**) and ORCA3 (**b**) in response to RN3 fungal elicitor and methyl jasmonate (MeJA). Eighteen-day-old Catharanthus roseus H01 suspension cells were treated with 1% RN3, 150 μM MeJA, or their combination. Error bars represent the standard error (SE) of three biological replicates. The different letters represent significant differences (*p* < 0.05) among treatments at each time point.

**Figure 5:**
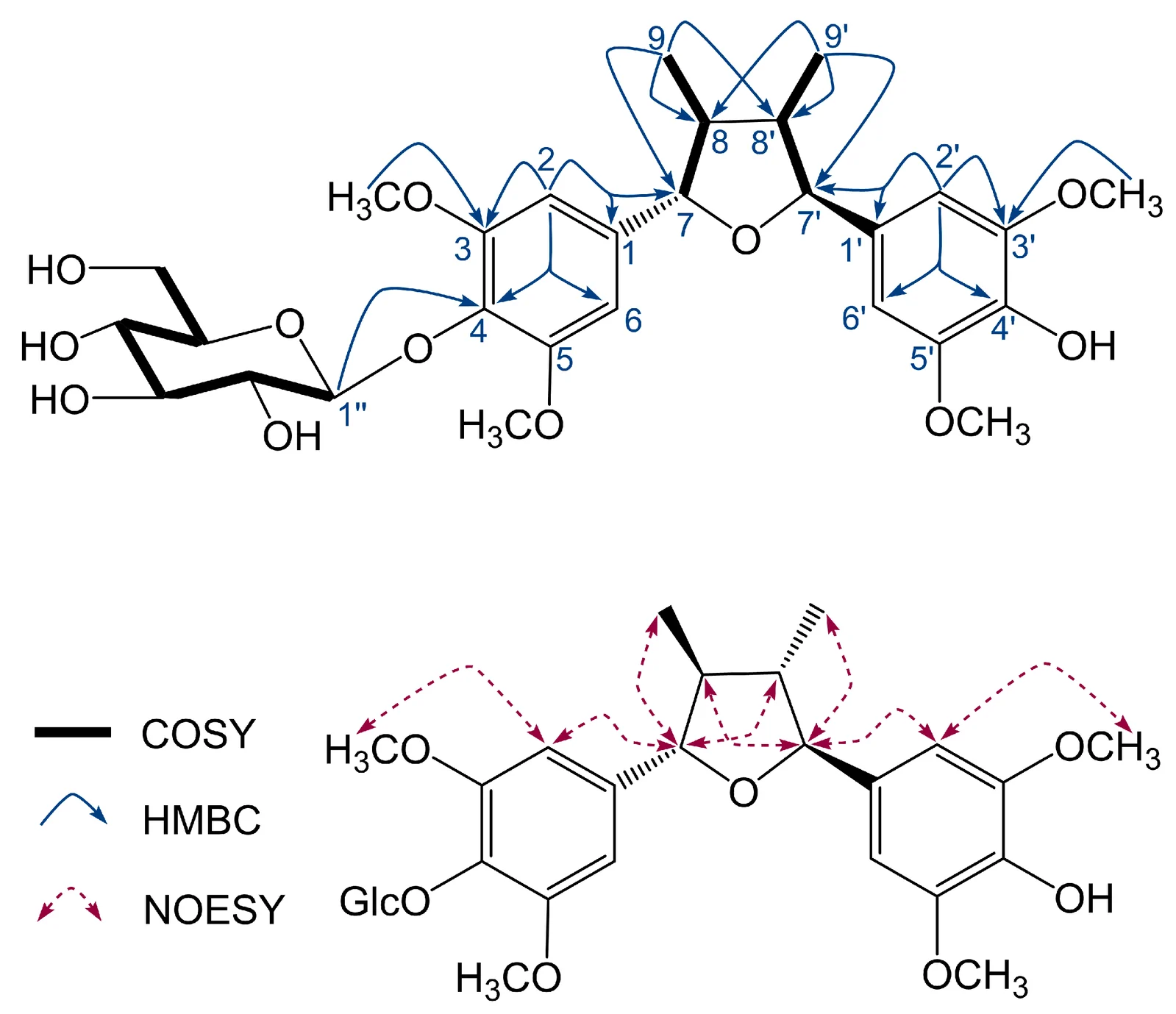
Key COSY, HMBC, and NOESY correlations of compound **1**.

## Data Availability

Data is contained within the article and the Supplementary Materials.

## Supplementary Materials

The following supporting information can be downloaded at https://www.mdpi.com/article/10.3390/plants15193051/s1, Figure S1: Structures of compounds **1**–**5**; Figure S2. ^1^H NMR spectrum of compound **1**: (A) CD_3_OD, 500 MHz; (B) CDCl_3_, 600 MHz; Figure S3: ^13^C NMR spectrum of compound **1**: (A) CD_3_OD, 125 MHz; (B) CDCl_3_, 150 MHz; Figure S4: HSQC spectrum of compound **1**: (A) CD_3_OD, 500 MHz; (B) CDCl_3_, 600 MHz; Figure S5: HMBC spectrum of compound **1**: (A) CD_3_OD, 500 MHz; (B) CDCl_3_, 600 MHz; Figure S6: COSY (CD_3_OD, 500 MHz) spectrum of compound **1**; Figure S7: NOESY (CD_3_OD, 500 MHz) spectrum of compound **1**; Figure S8: HR-ESI-MS spectrum of compound **1**; Figure S9: CD spectrum of compound **1**; Figure S10: Structural gene expression data. Table S1: List of primers used in this study.
