# Supplementary material for: Combined Fungal Elicitor and Methyl Jasmonate Enhance Alkaloid Accumulation in Catharanthus roseus Suspension Cultures: An Integrated Molecular and Phytochemical Study
Source: Plants (Basel). 2026 Oct 6;15(19):3051. doi: 10.3390/plants15193051 (PMC13666819; doi:10.3390/plants15193051)
Supplement: Supplementary file 1 [file plants-15-03051-s001.zip › Figure S10_Structural gene expression data.pdf]

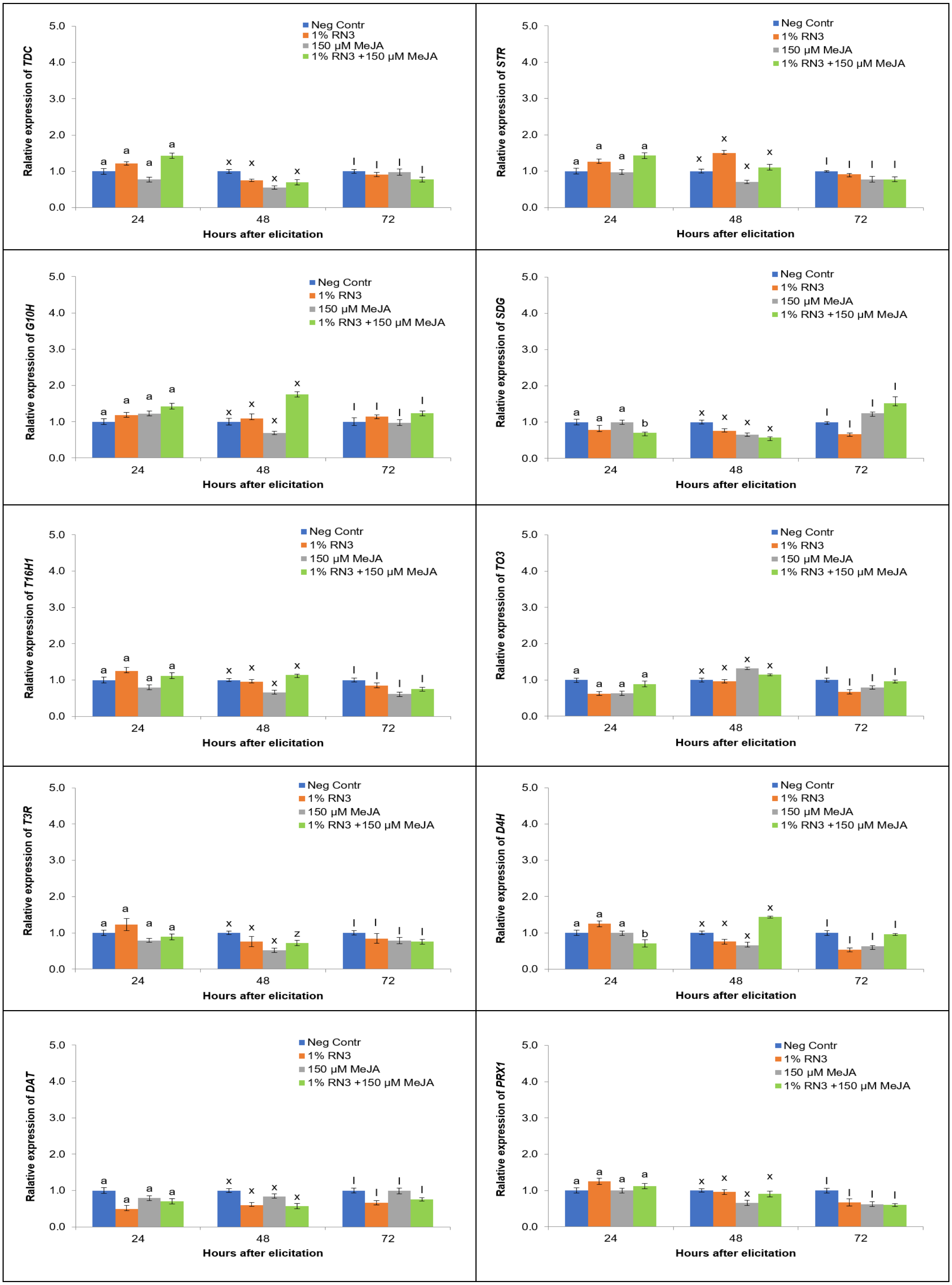

Figure S10. Structural gene expression data. *TDC* (Tryptophan decarboxylase), *STR* (Strictosidine synthase), *G10H* (Geraniol 10-hydroxylase), *SGD* (Strictosidine-O-beta-d-glucosidase), *T16H* (Tabersonine 16-hydroxylase), *T3O* (Tabersonine 3-oxygenase), *T3R* (tabersonine 3-reductase), *D4H* (Deacetoxyvindoline 4-hydroxylase), *DAT* (Deacetylvindoline O-acetyltransferase), and *PRX1* (Peroxidase 1) genes in *C. roseus* suspension cells following elicitation with RN3, MeJA, and their combination. Values sharing the same letter within each time point are not significantly different ($p > 0.05$).
