# Supplementary material for: Combined Fungal Elicitor and Methyl Jasmonate Enhance Alkaloid Accumulation in Catharanthus roseus Suspension Cultures: An Integrated Molecular and Phytochemical Study
Source: Plants (Basel). 2026 Oct 6;15(19):3051. doi: 10.3390/plants15193051 (PMC13666819; doi:10.3390/plants15193051)
Supplement: Supplementary file 1 [file plants-15-03051-s001.zip › Figures S1-S9.pdf]

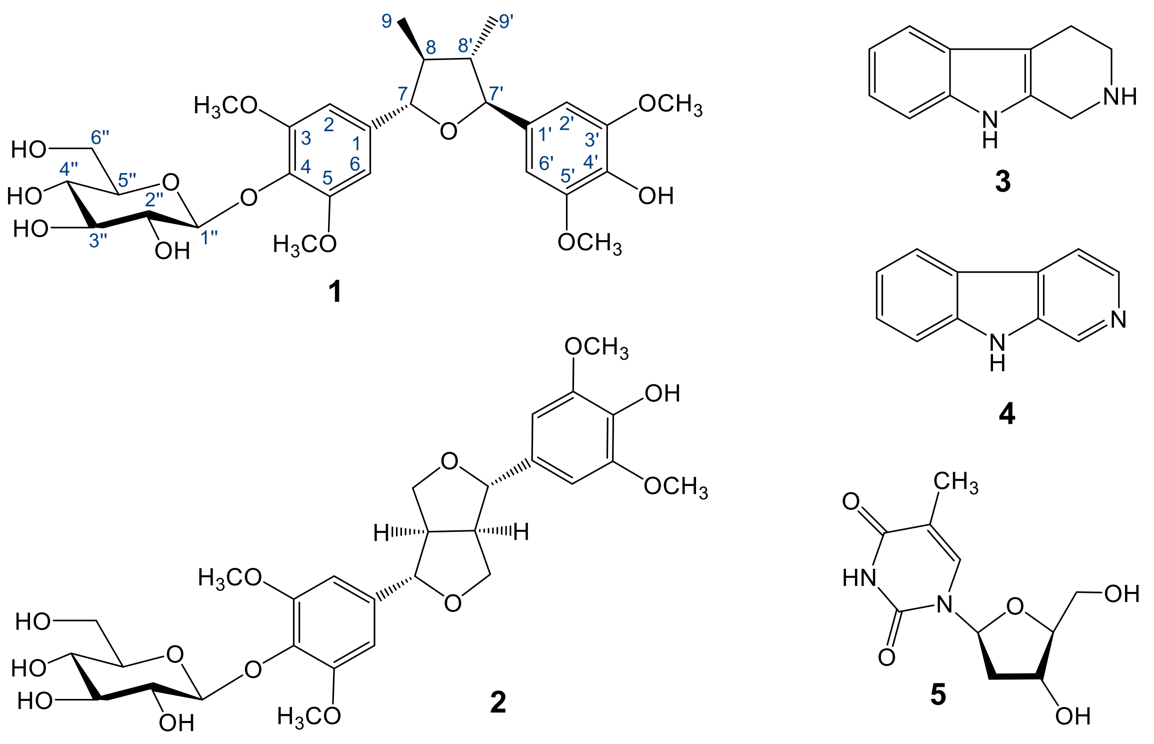

Figure S1. Structures of compounds **1**–**5**.

(A)

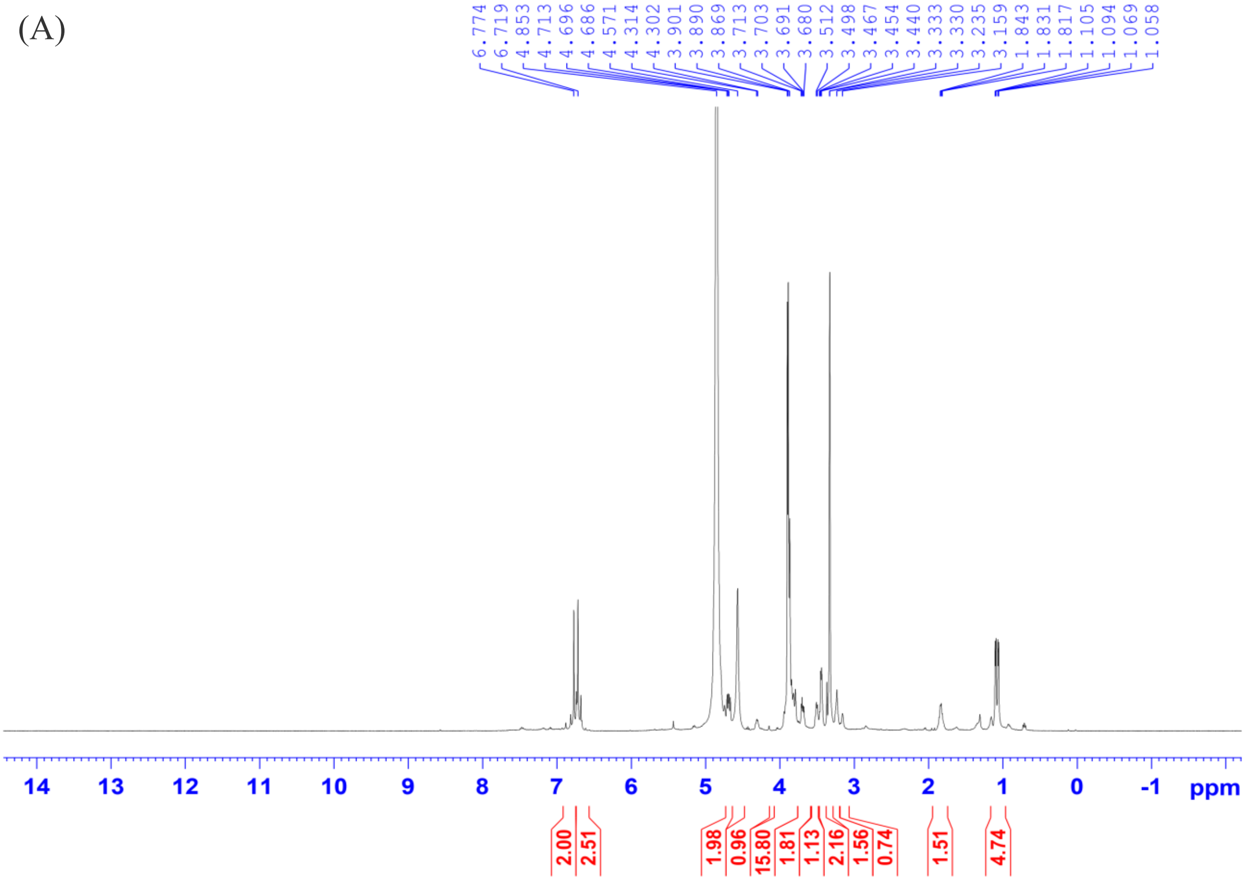

(B)

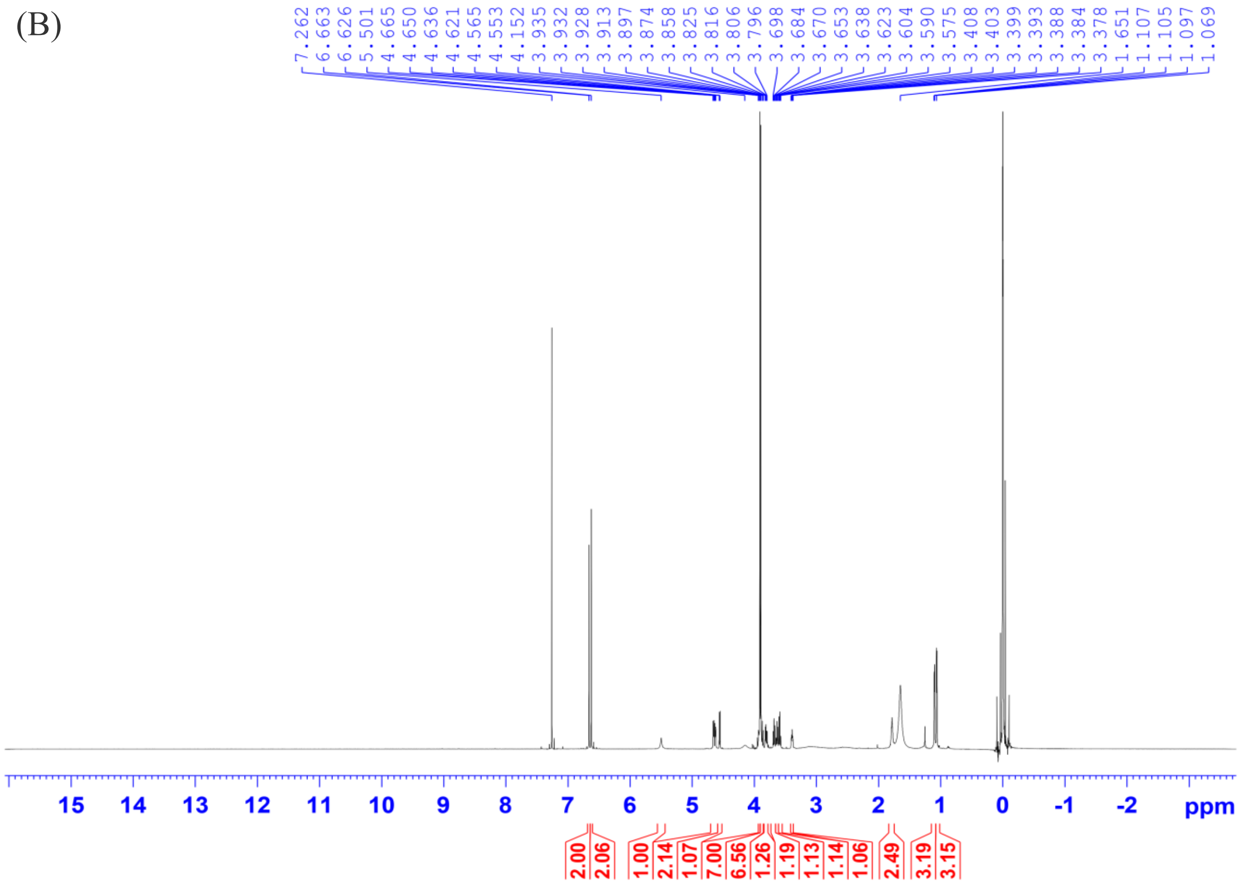

Figure S2. ^{1}H NMR spectrum of compound **1**: (A) CD_3OD, 500 MHz; (B) $CDCl_3$, 600 MHz.

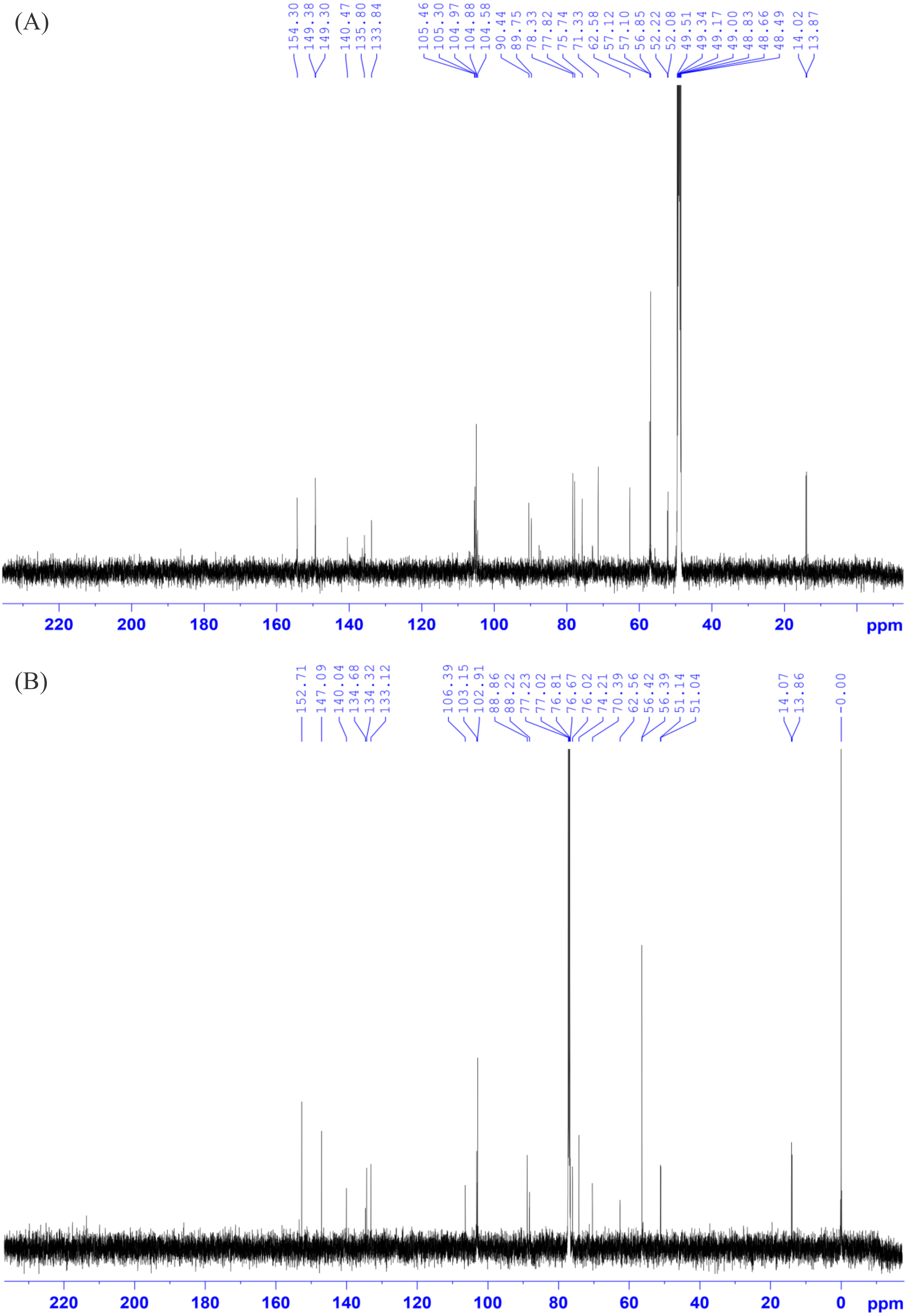

Figure S3. ^{13}C NMR spectrum of compound **1**: (A) CD_3OD, 125 MHz; (B) $CDCl_3$, 150 MHz.

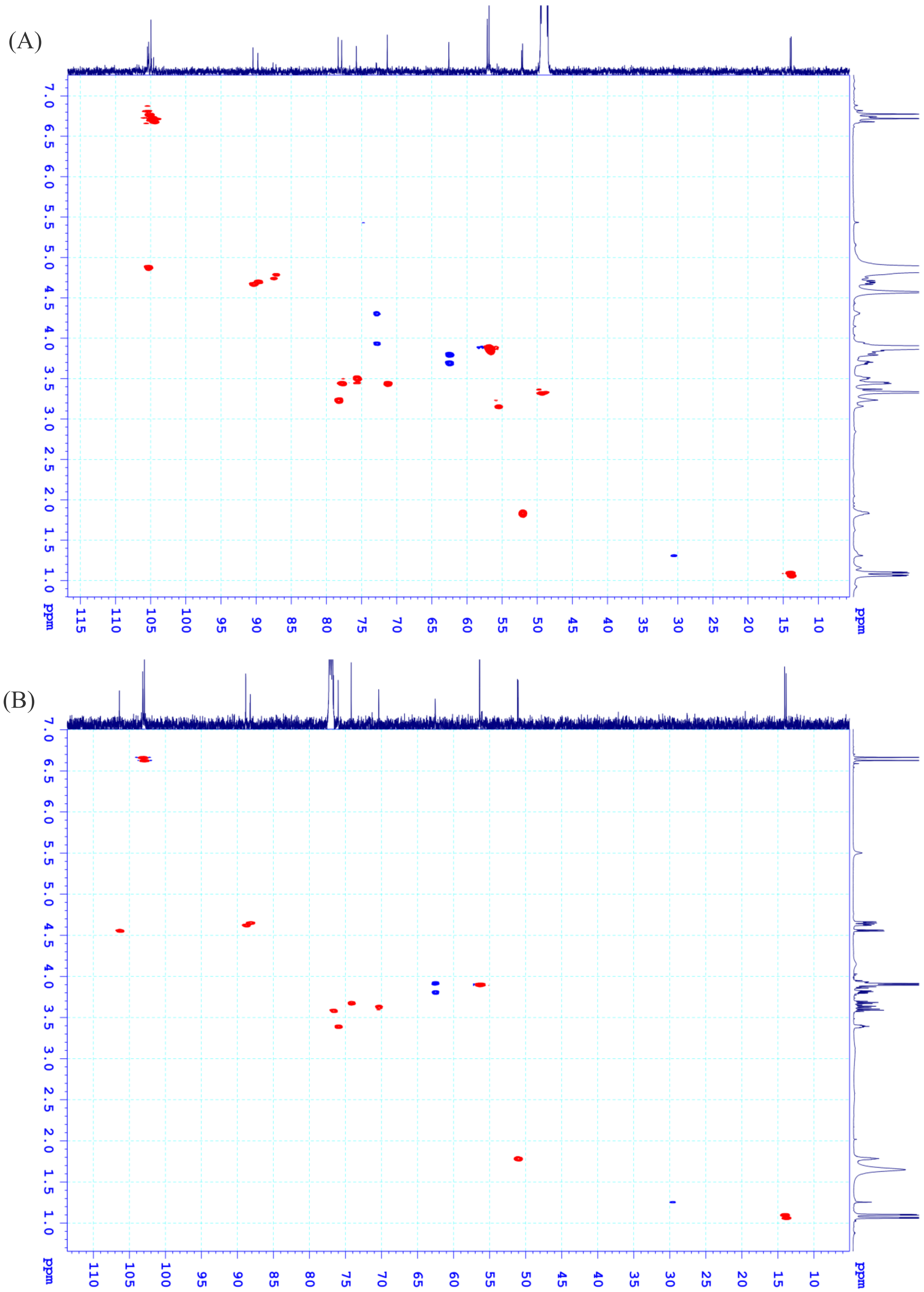

Figure S4. HSQC spectrum of compound **1**: (A) CD_3OD, 500 MHz; (B) $CDCl_3$, 600 MHz.

(A)

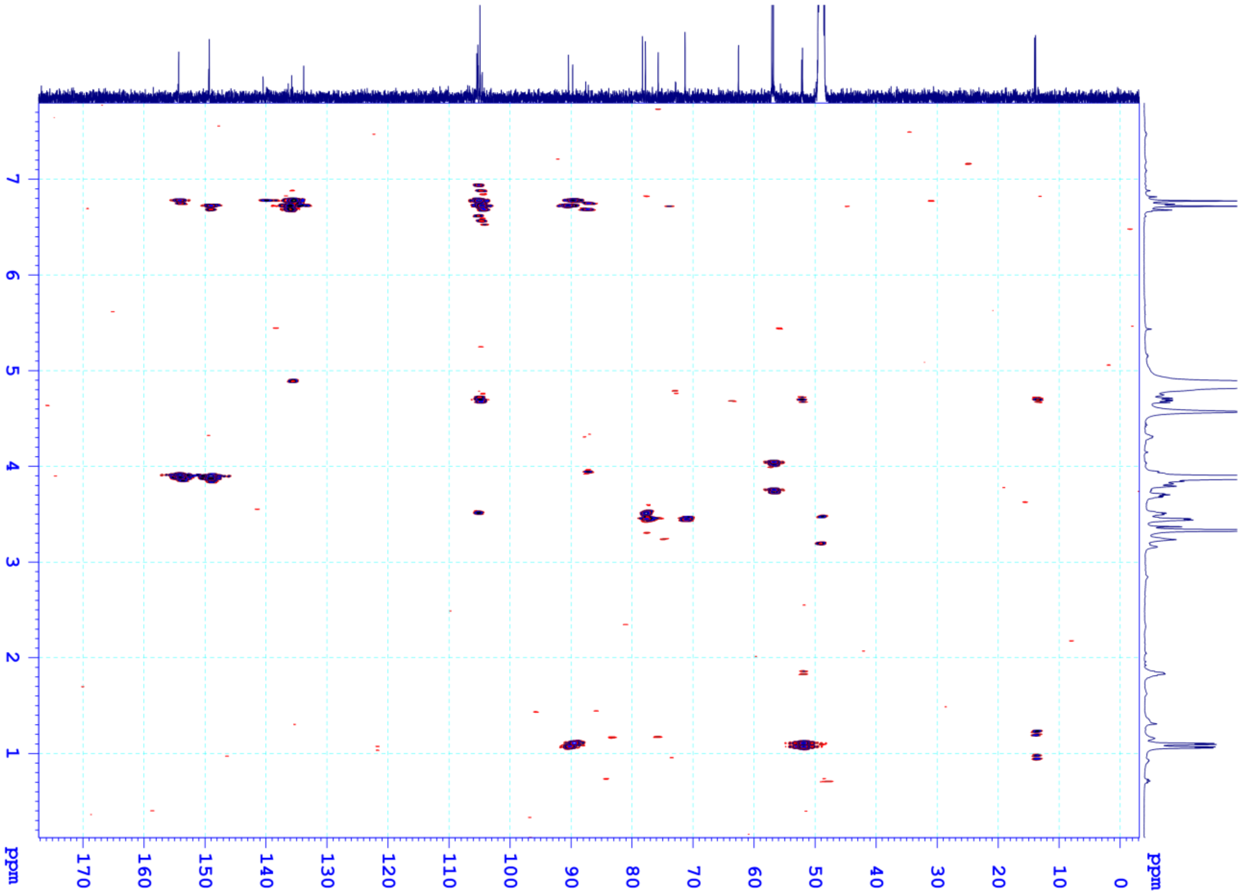

(B)

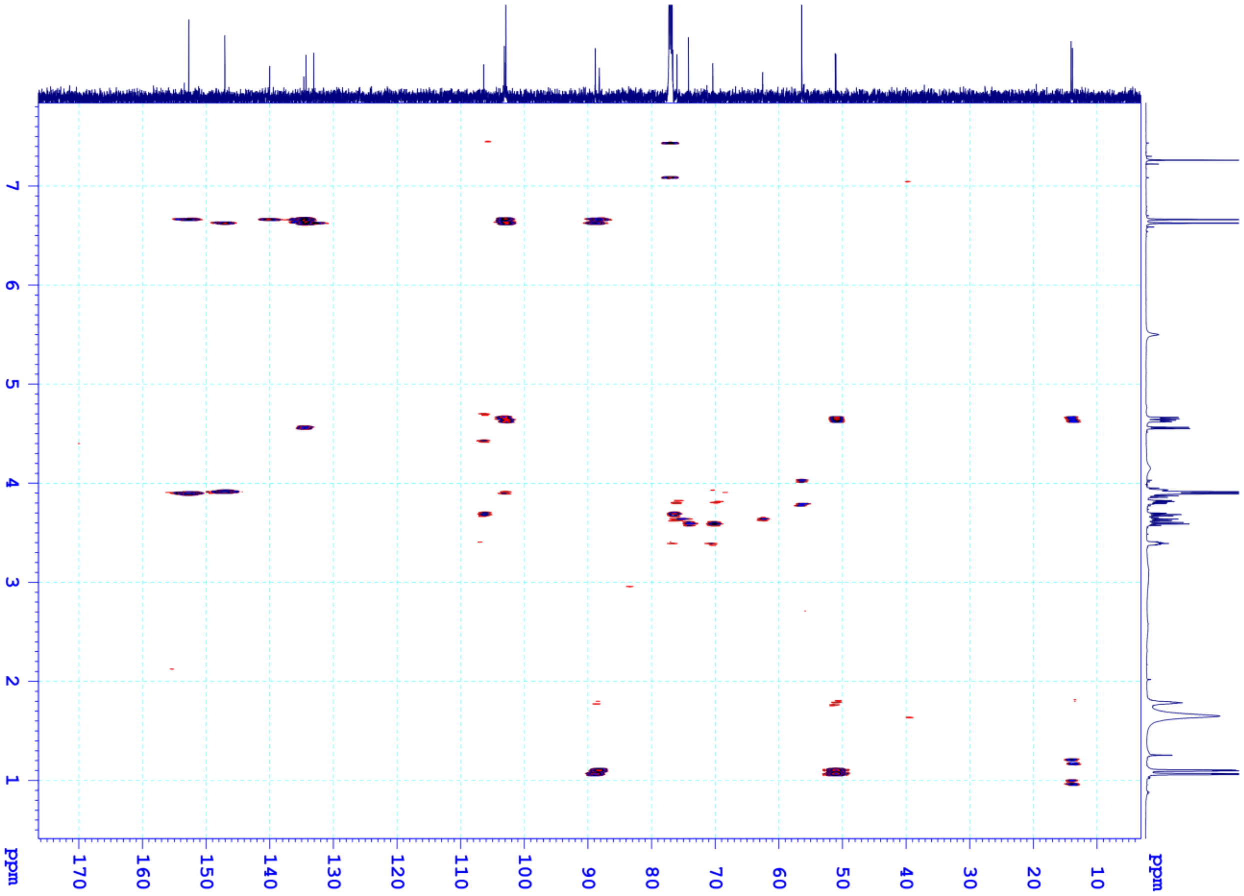

Figure S5. HMBC spectrum of compound **1**: (A) CD_3OD, 500 MHz; (B) $CDCl_3$, 600 MHz.

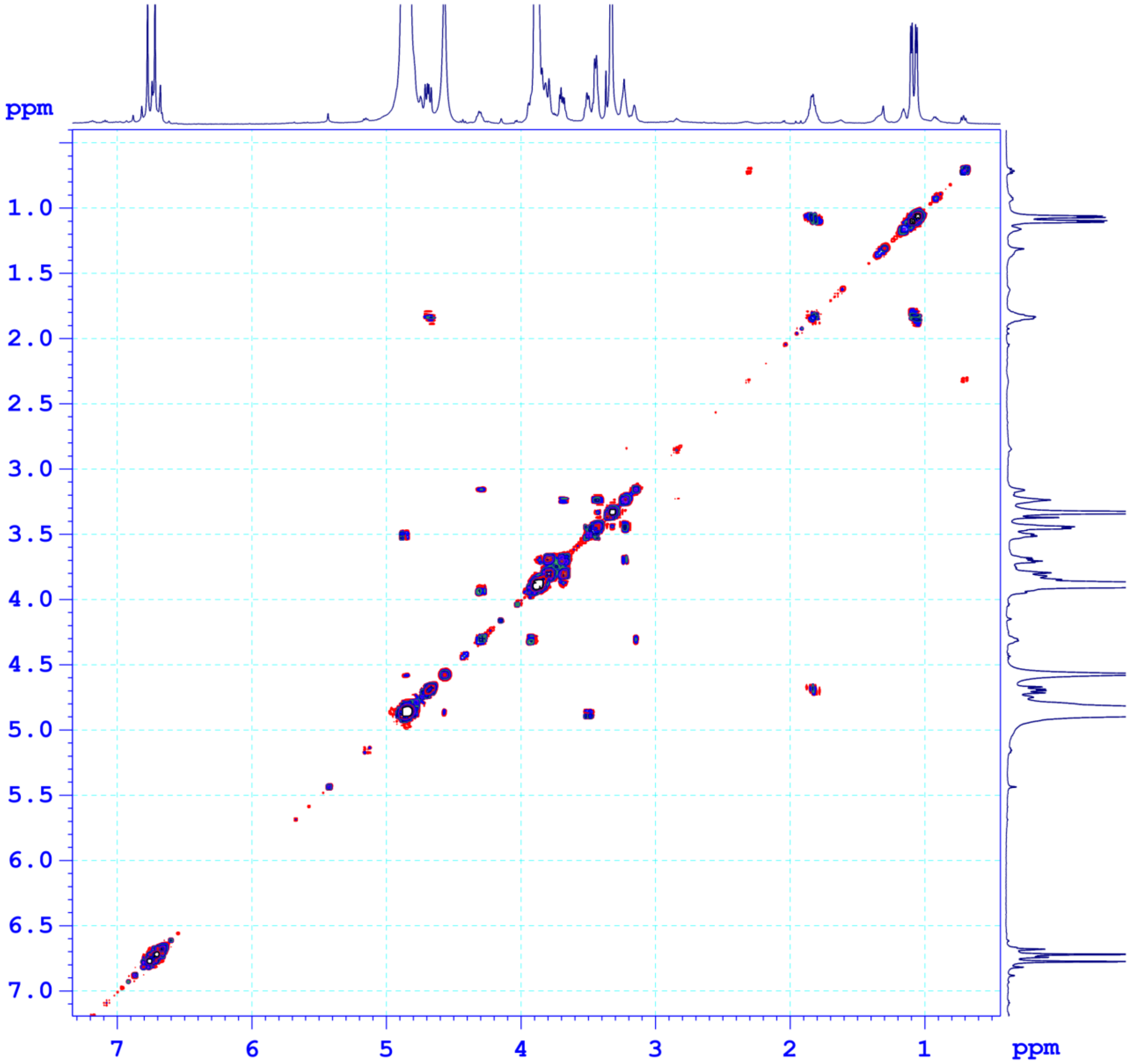

Figure S6. COSY (CD_3OD, 500 MHz) spectrum of compound **1**.

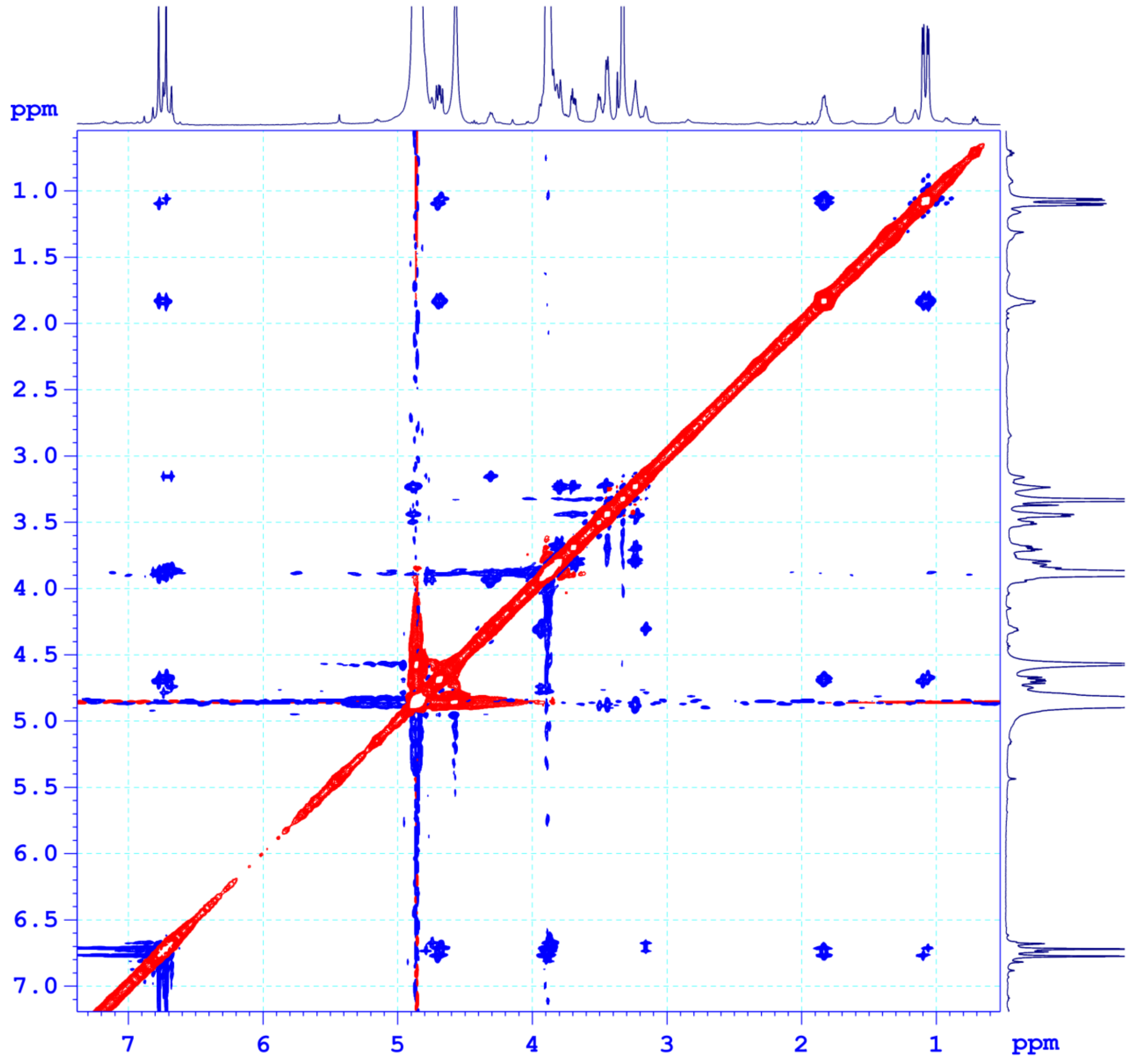

Figure S7. NOESY (CD_3OD, 500 MHz) spectrum of compound **1**.

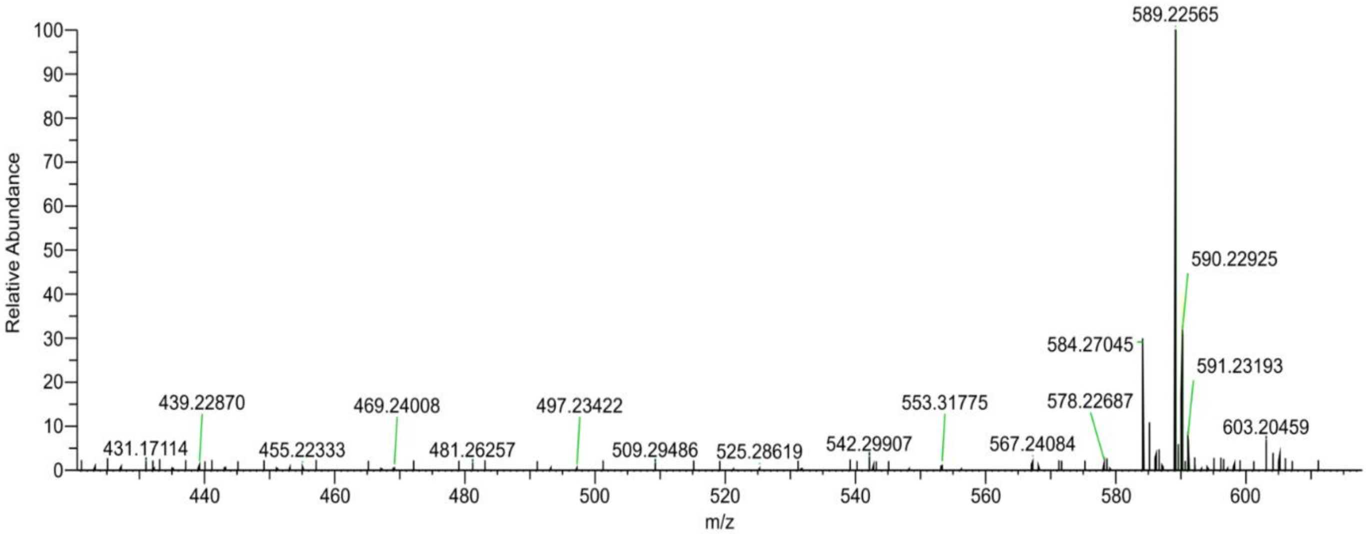

Peak Mass	Display For...	S Fit	RDB	Delta [mmu]	Theo. mass	Rank	Combined S...	# Matched I...	# Missed Iso.	MS Cov. [%]	Pattern Cov....	MSMS Matc...
589.22565	$C_{28}H_{38}O_{12}{}^{23}Na$	54.0702047312281	9.50	0.10	589.22555	1	94.21	4	1	96.44	99.88	(Collection)

Figure S8. HR-ESI-MS spectrum of compound **1**.

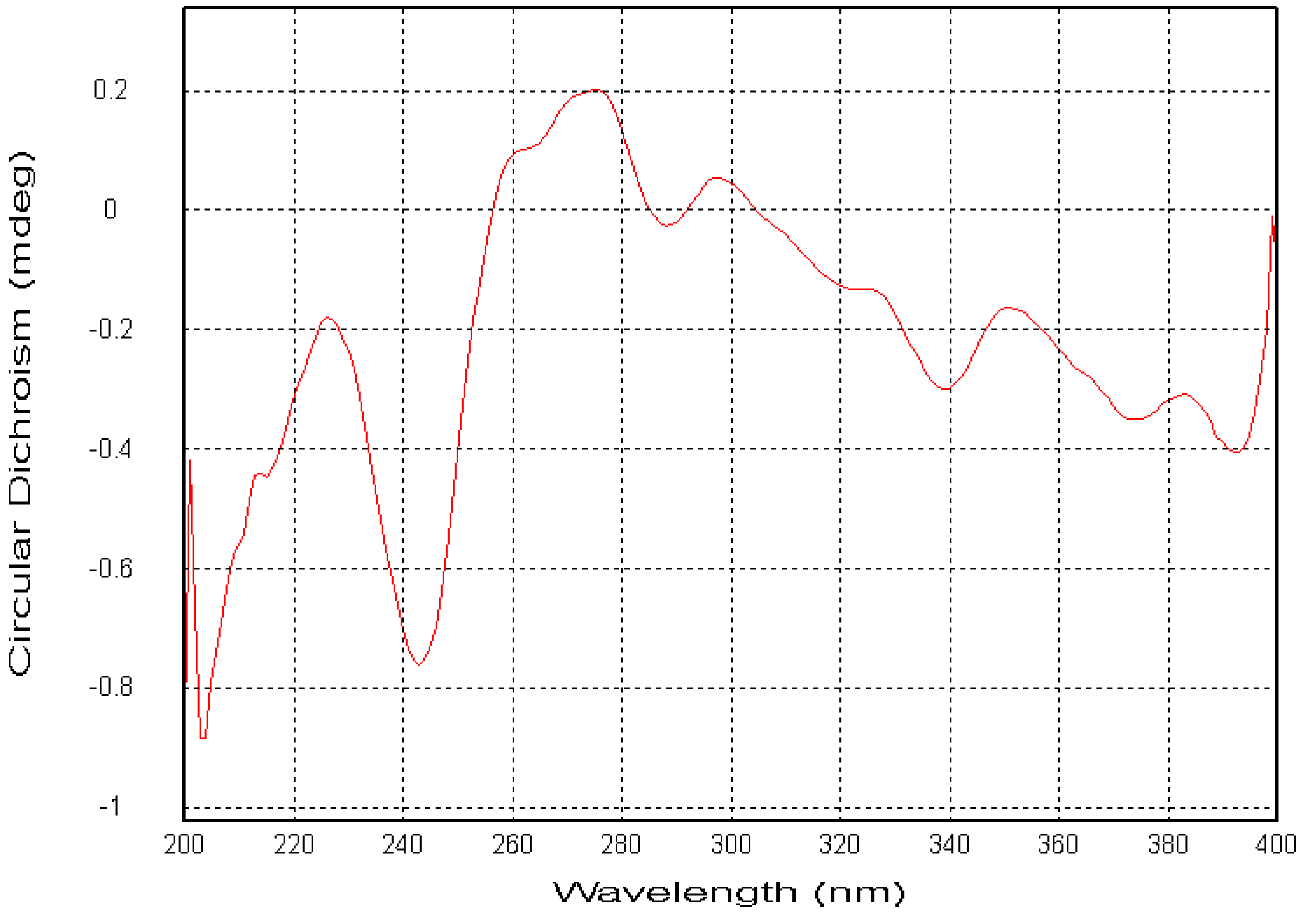

Figure S9. CD spectrum of compound **1**.
