# Supplementary material for: Combined Fungal Elicitor and Methyl Jasmonate Enhance Alkaloid Accumulation in Catharanthus roseus Suspension Cultures: An Integrated Molecular and Phytochemical Study
Source: Plants (Basel). 2026 Oct 6;15(19):3051. doi: 10.3390/plants15193051 (PMC13666819; doi:10.3390/plants15193051)
Supplement: Supplementary file 1 [file plants-15-03051-s001.zip › Table S1.pdf]

Table S1. List of primers used in this study.

No.	Genes	[1] Primer sequence (5′- 3′)	Amplicon length (bp)
1	*TDC* (Tryptophan decarboxylase)	CGAAATTGTGGTACCGAGAAAC<br>CGTCGAGTTAAGCATGTCCA	115
2	*STR* (Strictosidine synthase)	CGAAGGTGTGGAAGAAATCA<br>CGGGAACATGTAGCTCTTTC	110
3	*G10H* (Geraniol 10-hydroxylase)	ACAAGTAATCGGCAGAGGAA<br>TAAGAAGGGAACTGGTGGATG	112
4	*SGD* (Strictosidine-O-beta-d-glucosidase)	CCAGCCAAAATAGCTGATGG<br>ATTCCAAGCCTGTTTGCTTC	100
5	*T16H* (Tabersonine 16-hydroxylase)	GGAAGGGATCCAAATTACTGG<br>CCTTCTTCCACCACCAAATG	120
6	*T3O* (Tabersonine 3-oxygenase)	AAGCGTTCAATGGAAAGGAG<br>AATGGAGCAGGAGGGTGTAA	106
7	*T3R* (Tabersonine 3-reductase)	AAGAAGGGCTACAGGGGAAC<br>ACTTGGTCCTCCCGTATTTG	104
8	*D4H* (Deacetoxyvindoline 4-hydroxylase)	TCTGTTGCTGTTGCTTTTGG<br>AATGGGTGGATTTTCCTCTG	105
9	*DAT* (Deacetylvindoline O-acetyltransferase)	GCAGCTACAAAATTCGCTCTC<br>CGAACTCAATTCCATCGTCA	107
10	*ORCA2* (octadecanoid-responsive Catharanthus AP2-domain protein 2)	CGTTTCAACTCCGTGGTTCT<br>AGGACTTACTCTAACCGGAGCA	83
11	*ORCA3* (octadecanoid-responsive Catharanthus AP2-domain protein 3)	CGCGTTTAATATGCGTGGAG<br>ACTCTAACGGGTCCGGAAAT	81
12	*PRX1* (Peroxidase 1)	CATGTCCAACACCAGATTCA<br>CTTGGTCAGAAGTGAAAAGTCC	120
13	*RPS9* (40S ribosomal protein S9)	GAGCTTTGGAGGGTTCAATATG<br>TGGGTCCTTCTCATCCAAAG	81

[1] All primers were designed in this study.
